# Quantitative proteomic and phosphoproteomic analysis reveal the relationship between mitochondrial dysfunction and cytoskeletal remodeling in hiPSC-CMs deficient in PINK1

**DOI:** 10.1186/s12967-023-04467-y

**Published:** 2023-08-30

**Authors:** Huiwen Liu, Li Wang, Hao Xu, Bin Tan, Qin Yi, Hongrong Deng, Yunxia Chen, Bolin He, Jie Tian, Jing Zhu

**Affiliations:** 1 Ministry of Education Key Laboratory of Child Development and Disorders, Department of Pediatric Research Institute, National Clinical Research Center for Child Health and Disorders, Children’s Hospital of Chongqing Medical University, Chongqing, China; 2 Chongqing Key Laboratory of Pediatrics, Children’s Hospital of Chongqing Medical University, Chongqing, China; 3 Department of Clinical Laboratory, Children’s Hospital of Chongqing Medical University, Chongqing, China; 4 Department of Blood Transfusion, Children’s Hospital of Chongqing Medical University, Chongqing, China; 5 Department of Cardiovascular (Internal Medicine), Children’s Hospital of Chongqing Medical University, Chongqing, China

**Keywords:** hiPSC-CMs, PINK1, Proteomic, Phosphoproteomic, Cytoskeleton, Mitochondrial, Respiratory chain

## Abstract

**Background:**

Human induced pluripotent stem cell-derived cardiomyocytes (hiPSC-CMs) are seed cells that can be used for alternative treatment of myocardial damage. However, their immaturity limits their clinical application. Mitochondrial development accompanies cardiomyocyte maturation, and PINK1 plays an important role in the regulation of mitochondrial quality. However, the role and mechanism of PINK1 in cardiomyocyte development remain unclear.

**Methods:**

We used proteomic and phosphoproteomic to identify protein and phosphosite changes in hiPSC-CMs deficient in PINK1. Bioinformatics analysis was performed to identify the potential biological functions and regulatory mechanisms of these differentially expressed proteins and validate potential downstream mechanisms.

**Results:**

Deletion of PINK1 resulted in mitochondrial structural breakdown and dysfunction, accompanied by disordered myofibrils arrangement. hiPSC-CMs deficient in PINK1 exhibited significantly decreased expression of mitochondrial ATP synthesis proteins and inhibition of the oxidative phosphorylation pathway. In contrast, the expression of proteins related to cardiac pathology was increased, and the phosphoproteins involved in cytoskeleton construction were significantly altered. Mechanistically, PINK1 deletion damaged the mitochondrial cristae of hiPSC-CMs and reduced the efficiency of mitochondrial respiratory chain assembly.

**Conclusion:**

The significantly differentially expressed proteins identified in this study highlight the important role of PINK1 in regulating mitochondrial quality in hiPSC-CMs. PINK1-mediated mitochondrial respiratory chain assembly is the basis for mitochondrial function. Whereas the cytoskeleton may be adaptively altered in response to mitochondrial dysfunction caused by PINK1 deletion, inadequate energy supply hinders myocardial development. These findings facilitate the exploration of the mechanism of PINK1 in cardiomyocyte development and guide efforts to promote the maturation of hiPSC-CMs.

**Supplementary Information:**

The online version contains supplementary material available at 10.1186/s12967-023-04467-y.

## Introduction

Cardiovascular disease continues to be the foremost cause of mortality worldwide [1]. Conventional pharmacotherapy primarily focuses on reducing cardiac workload without halting disease progression, leading to a suboptimal prognosis for patients with cardiovascular diseases. Moreover, alternatives to pharmacological interventions, such as organ transplants and mechanical support devices, are often accompanied by considerable risks of morbidity and death. Hence, the identification of effective treatment strategies for cardiovascular disease is of paramount importance.

Human induced pluripotent stem cells (hiPSCs) have remarkable multidirectional differentiation capability, including their capacity to differentiate into functional beating cardiomyocytes within a short timeframe [2]. However, current human induced pluripotent stem cell-derived cardiomyocytes (hiPSC-CMs) exhibit morphological and functional similarities to fetal cardiomyocytes [3], limiting their full clinical utility due to inadequate integration into damaged heart tissue [4]. During fetal development, the heart is constantly exposed to changing levels of oxygen, nutrients, and hormones, which drive metabolic shifts crucial to cardiac maturation [5]. Among the key players in this process are mitochondria, which not only adapt to these metabolic changes but also actively promote cardiomyocyte maturation. Therefore, unraveling the intricate mechanisms through which mitochondria influence cardiomyocyte maturation during development and identifying actionable targets are of paramount importance. This will help increase the feasibility of utilizing maturation cardiomyocyte for clinical applications.

The heart, a mitochondria-rich organ, relies on mitochondrial homeostasis for proper cardiac development. Mitochondrial function, including the rapid delivery of metabolites and ions, is closely tied to the structure and distribution of mitochondrial membranes [6]. PTEN-inducible kinase 1 (PINK1), a serine/threonine kinase, a highly conserved gene associated with Parkinson’s disease susceptibility [7]. It is ubiquitously expressed and localized in mitochondria and plays an important role in mitochondrial integrity [8]. Recessive genetic mutations in PINK1 disrupt mitochondrial dynamics and quality, ultimately leading to disease or developmental disorders [9–12]. Although the importance of PINK1 in the regulation of mitochondrial quality is well established, the precise molecular mechanisms underlying PINK1 regulation remain elusive.

In this research, we conducted high-throughput screening to identify differentially expressed proteins and phosphosites associated with the deletion of PINK1 in hiPSC-CMs. Our findings demonstrated that these differentially expressed proteins were differentially localized and engaged in various biological processes. Specifically, the downregulated proteins were localized in the mitochondria and played a role in ATP synthesis and oxidative phosphorylation, while the upregulated proteins were primarily found in focal adhesion, the extracellular matrix, and the cytoskeleton and were implicated in cytoskeletal organization and cardiac pathological processes. Furthermore, motif analysis was used to identify potential upstream kinases connected with the differentially phosphosites. These predicted kinases were associated with cytoskeleton organization and energy metabolism. Additionally, we found that deletion of PINK1 resulted in impaired assembly of the respiratory chain, forcing remodeling of hiPSC-CMs in response to low energy availability. In conclusion, our work elucidates the role of PINK1 in cardiomyocyte developmental maturity and provides insights into downstream signaling pathways that may be influenced by PINK1 during hiPSC-CMs development, thus offering potential downstream targets for therapeutic intervention.

## Methods

### Culture of hiPSCs and differentiation into cardiomyocytes

The hiPSCs employed in this study were reprogrammed from human urine-derived kidney epithelial cells (CELLAPY, China). The hiPSCs were cultured on matrix gel-coated plates using PGM1 medium (CELLAPY, China). Then, 6 μM CHIR (Selleck, USA) was added to B27 supplement lacking insulin (Thermo Fisher Scientific, USA)-RPMI 1640 (Sigma, USA) at day 0. After 48 h, the medium was changed to B27 supplement lacking insulin-RPMI 1640. Then, 5 μM IWP2 (Selleck, USA) was added to the B27 supplement lacking insulin-RPMI 1640 at day 3. After 48 h, the media was changed to B27 supplement lacking insulin-RPMI 1640. B27 supplement with insulin (Thermo Fisher Scientific, USA) was added at day 7. At day 13, the cells were purified B27 with insulin-glucose-free RPMI 1640 (Thermo Fisher Scientific, USA) containing 4 mM lactate (Sigma, USA). At day 16, the medium was replaced with B27 supplement with insulin-RPMI 1640 for further culture. At day 18, cardiomyocytes were reinoculated and subsequently maintained using B27 supplement with insulin-RPMI 1640.

### siRNA transfection and drug treatment

According to the manufacturer’s instructions, siRNA (RIBOBIO, China) and RNAiMAX (Thermo Fisher Scientific, USA) were diluted in Opti MEM. The diluted siRNA and RNAiMAX were mixed at a 1:1 dilution and allowed to stand for 5 min. Subsequently, the mixture was added to cells for transfection. In this experiment, the concentration of siPINK1 utilized was 100 nM.

We used 1 μM kinetin (MCE, China) as the experimental treatment concentration. After siPINK1 treatment of hiPSC-CMs for 6 h, the medium was replaced with fresh medium, kinetin was added, and the culture was continued until 48 h for subsequent experiments.

### Proteome and phosphoproteome analyses

#### Sample preparation

Cell samples were lysed using high-intensity ultrasonic processing in a lysis buffer. The supernatant was collected by centrifugation, and the protein concentration was measured using a BCA kit.

#### Protein digestion and TMT labeling

Protein solutions were reduced for 30 min at 56 °C with 5 mM dithiothreitol and then alkylated for 15 min at room temperature with 11 mM iodoacetamide, protected from light. Next, the protein samples were diluted with 100 mM TEAB to reach a urea concentration less than 2 M. Protein digestion was performed overnight using trypsin. The pooled samples were desalted on a Strata X C18 SPE column and vacuum centrifuged to dry. Subsequently, tryptic peptides were dissolved in 0.5 M TEAB and labeled for 2 h at room temperature with the respective TMT reagent (Thermo Fisher Scientific, USA). To determine the labeling efficiency, each labeled sample was desalted and analyzed by MS. Following confirmation of labeling efficiency, the samples were quenched with 5% hydroxylamine.

#### Phosphorylated peptide enrichment

The peptide mixture was shaken in loading buffer (50% acetonitrile/0.5% acetic acid) with IMAC microsphere suspension. IMAC microspheres were washed sequentially with 50% acetonitrile/0.5% acetic acid and 30% acetonitrile/0.1% trifluoroacetic acid. Phosphopeptide enrichment samples were eluted with 10% NH_4_OH elution buffer while shaking. The phosphopeptide-containing supernatant was collected, lyophilized, and subjected to LC–MS/MS analysis.

#### LC–MS/MS analysis

The enriched phosphorylated peptides were separated using an Agilent 300 Extend C18 column on an EASY-nLC 1200 UPLC system (Thermo Fisher Scientific, USA). The electrospray voltage was set at 2.0 kV, and the scan resolution for complete mass spectrometry was set to 60,000 with a scan range of 350–1600 m/z. With a dynamic exclusion time of 30 s, up to 20 of the most abundant precursors were selected for subsequent LC–MS/MS analysis. HCD fragmentation was carried out at a normalized collision energy (NCE) of 28%, and the fragments were identified on the Orbitrap at a resolution of 30,000. The automatic gain control (AGC) target was set to 1E5, the intensity threshold was set to 3.3E4, and the maximum injection duration was set to 60 ms.

#### Database search and data analysis

The LC–MS/MS data obtained were analyzed using the MaxQuant search engine (v.1.6.15.0). Tandem mass spectrometry was searched against the human SwissProt database (20,422 entries) and the reverse decoy database for peptides, proteins, and modified peptides, using a false discovery rate (FDR) threshold of < 1%. TMT-labeled proteomic analysis was also carried out in the MaxQuant environment. All further data supporting the conclusions can be found in the article and Additional files.

#### Bioinformatics analysis

The Database for Annotation, Visualization, and Integrated Discovery (DAVID) Bioinformatics Resources 6.747 was used to conduct the Gene Ontology (GO) enrichment analysis [13, 14]. Subsequently, Kyoto Encyclopedia of Genes and Genomes (KEGG) enrichment analysis was performed online using Metascape (http://metascape.org) [15]. Protein–protein interaction (PPI) networks were analyzed using the STRING database (https://string-db.org) [16], with a composite score > 0.7 indicating statistically significant interactions. The results of this analysis were visualized using Cytoscape 3.9.0 software.

Motif-X algorithm analysis software (http://motif-x.med.harvard.edu/) was used in this study to find kinase-specific phosphosites and to determine the interaction between kinases and substrates. Peptide sequences comprising the 6 amino acids upstream and downstream of the modification sites were used as the target of analysis, with all peptide sequences in the species serving as the background for analysis. Only those peptides with > 20 occurrences of characteristic sequences and a statistical test *P* < 0.000001 were considered. iGPS 1.0 software was used to predict kinase-substrate regulation, while the GPS 2.0 algorithm was employed to predict kinase-substrate relationships at specific sites. Additionally, GSEA 4.0.3 software (http://software.broadinstitute.org/gsea/index.jsp) was used to predict kinase activity, with enrichment significance based on a *P* < 0.05 and FDR < 0.25 as the criterion.

### Immunofluorescence staining of hiPSC-CMs

Cardiomyocytes were given a fixation of 20 min in 4% paraformaldehyde and three times PBS washed thereafter. 0.5% Triton X-100 was applied for 10 min to permeabilize the cells. The cells were then blocked for 30 min at room temperature with 5% bovine serum albumin. The cells were then treated with the primary antibody dilutions for 2 h at 37 °C in an incubator before being washed three times with PBS. Subsequently, the cells were treated with secondary antibody dilutions or ActinTracker working solution (1:100; Beyotime, China) for 1 h at 37 °C in an incubator before being washed three times with PBS. After blocking with an anti-fluorescence quencher, the slices were photographed with either an A1 confocal microscope or a 90I inverted fluorescence microscope. Table supplement in Additional file 1, the details of the antibody catalogs used in this study are presented.

### MitoTracker staining

A working solution of MitoTracker (1 mM; Beyotime, China) was prepared. After discarded the medium in the confocal dishes, PBS was used to wash them. Then, 950 μl of MitoTracker working solution was added, and the samples were incubated at 37 °C for 15 min. The dishes were washed three times with PBS after the working solution was removed. Finally, 1 ml of fresh medium was added, and an A1 confocal microscope was used to photograph.

### Detection of mitochondrial membrane potential (ΔΨm)

The JC-1 probe (Beyotime, China) was used to measure mitochondrial membrane potential. The JC-1 staining working solution was prepared by diluting 50 μl of JC-1 (200X) in 8 ml of ultrapure water, then adding 2 ml of JC-1 staining buffer (5×). Following incubation, 2 ml of fresh medium was added after the cells had been washed with JC-1 staining buffer (1×), and an A1 confocal microscope was used to photograph.

### Assay of cellular ATP content

The ATP test kit (Beyotime, China) was used to determine the cellular ATP content. The lysate was added to the culture plate and the cells were lysed on ice. After lysis, the supernatant was collected by centrifugation at 4 °C for 5 min at 12,000×*g*. A Cytation 5 microplate reader (Biotek, USA) was used to determine the relative light unit (RLU) values.

### Cellular reactive oxygen species (ROS) assay

At a concentration of 1:1000, serum-free medium was added with DCFH-DA (10 mM; Beyotime, China). After removing the original cell culture medium and adding the proper amount of DCFH-DA dilution, the samples were incubated at 37 °C for 20 min. The cells were washed three times with serum-free medium. A Cytation 5 microplate reader (Biotek, USA) was used to photograph.

### Transmission electron microscopy

The samples were infiltrated with Epox 812 for an extended period, embedded, dehydrated in acetone, postfixed with 1% osmium tetroxide, and fixed with 3% glutaraldehyde. Methylene blue was used to stain semithin sections, and ultrathin sections were then stained with uranyl acetate and lead citrate. A JEM-1400-FLASH transmission electron microscope was used to examine the sections.

### RNA extraction and quantitative PCR

According to the manufacturer’s instructions, RNA from hiPSC-CMs was extracted using the TRIzol method, followed by Takara Reverse Reagent (TaKaRa, Japan) for reverse transcription to cDNA. TB Green (TaKaRa, Japan) and the Bio-Rad Real-Time Quantification System (USA) were then used to quantify the cDNA. All samples were subjected to semiquantitative analysis using the ∆∆Ct method with three biological replicates. Table supplement in Additional file 1 contains details of the primer catalog used in this study.

### *Western blotting and co-immunoprecipitation (Co*-*IP)*

BCA assay kit (KeyGen Biotech, China) was used to quantify the concentration of proteins that were extracted from hiPSC-CMs using lysis buffer. Electrophoresis was used to separate protein samples, which were then transferred to PVDF membranes (Millipore Sigma, China). The primary antibodies were incubated overnight at 4 °C after the membranes had been blocked with skim milk. An ECL imaging system (Bio-Rad, USA) was used to visualize the primary antibodies after they had been conjugated with HRP-conjugated IgG secondary antibody.

Co-IP assays were performed according to the manufacturer’s instructions of the Co-IP kit (Absin, China). Primary antibody (2.5 μg) and cell lysates were mixed overnight at 4 °C. The cell lysates were subsequently mixed with Protein A and Protein G at 4 °C for 3 h. The samples were then centrifuged and washed, and the precipitate was retained. Finally, 30 µl of 1× SDS buffer was added and then boiled, and the supernatant was collected for western blotting after centrifugation.

### Statistical methods

Morphological analysis was performed using ImageJ, with the Manders overlap coefficient (MOC) calculated using JACoP [17]. Three biological replicates of each experiment were carried out. GraphPad Prism 9.0 was used to analyze the data and create graphs. Statistical analysis between groups was conducted using the unpaired Student’s t test, and the Mann–Whitney test was used when data were not normally distributed. A significance level of *P* < 0.05 was considered statistically significant, and the mean ± standard deviation was used to represent the data.

## Results

### PINK1 regulates the mitochondrial quality and contributes to hiPSC-CMs development

hiPSCs were differentiated into cardiomyocytes using temporal modulation of the Wnt signaling pathway [2], as illustrated in Additional file 3: Fig. S1A. Immunofluorescence analysis confirmed the expression of the NANOG, SOX2, and OCT4 stemness markers in hiPSCs (Additional file 3: Fig. S1B), while α-actinin, TNNT2, and CX43 cardiomyocyte markers were expressed in hiPSC-CMs (Additional file 3: Fig. S1C). Ultrastructural analysis by electron microscopy revealed that hiPSCs contained a small number of short spherical mitochondria, whereas hiPSC-CMs possessed abundant myofilaments and elongated rod-shaped mitochondria (Additional file 3: Fig. S1E). These results collectively demonstrate the successful generation of hiPSC-CMs in our study.

**Fig. 1:**
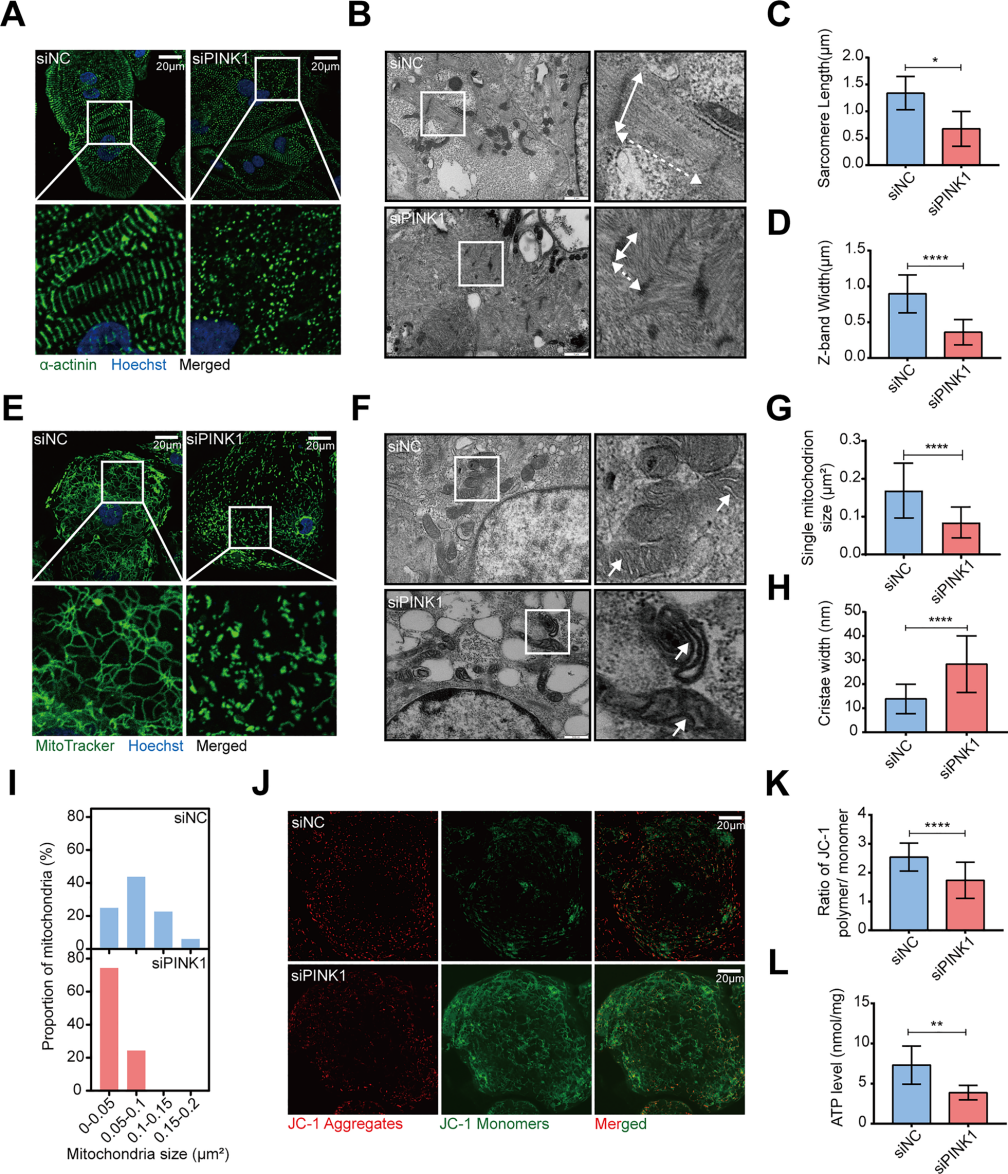
PINK1 deletion reduced mitochondrial quality and inhibited hiPSC-CMs maturation. **A** α-actinin staining of hiPSC-CMs with inhibited PINK1 expression. α-actinin (green), Hoechst (blue). Scale bar = 20 μm. **B** Electron microscopy images of hiPSC-CMs with inhibited PINK1 expression. z-band (solid arrow), sarcomere (dashed arrow). ImageJ assay sarcomere length (**C**) and Z-band width (**D**). Scale bar = 1 μm. **E** MitoTracker staining of hiPSC-CMs that inhibit PINK1 expression. MitoTracker (green), Hoechst (blue). Scale bar = 20 μm. **F** Electron microscopy images of hiPSC-CMs that inhibit PINK1 expression. The white arrows point to the intercristal space of mitochondria. ImageJ was used to examine the mitochondrial area (**G**) and cristae width (**H**) and determine the frequency of distribution of mitochondria of different sizes (**I**). Scale bar = 500 nm. **J** Mitochondrial membrane potential of hiPSC-CMs inhibiting PINK1 expression, JC-1 aggregates (red), JC-1monomers (green). Scale bar = 20 μm. **K** Statistical plot of the mitochondrial membrane potential of hiPSC-CMs inhibiting PINK1 expression. **L** ATP content of hiPSC-CMs inhibiting PINK1 expression. The means ± SEMs are shown. *P < 0.05, **P < 0.01, ***P < 0.001, ****P < 0.0001

To investigate the role of PINK1 in cardiomyocyte maturation, we employed siRNA to inhibit the expression of PINK1 in hiPSC-CMs (Additional file 4: Fig. S2A). Sarcomeres, which are fundamental units of cardiomyocyte contraction, are highly dependent on tissue structural arrangement for proper function [18]. Immunofluorescence analysis revealed that while both the siNC group and siPINK1 group expressed α-actinin, the sarcomeres in the siPINK1 group were shortened and disorganized than those of the siNC group (Fig. 1A). Ultrastructural analysis by electron microscopy revealed that the myofibrils of the siPINK1 group were disorganized (Fig. 1B) and had shorter Z-bands and sarcomeres (Fig. 1C, D). Cardiomyocyte development involves metabolic transformations [19, 20], and mitochondria, which serve as the primary source of energy, are crucial for maintaining energy production [21]. Our results demonstrated that knockdown of PINK1 caused the fragmentation of mitochondria in hiPSC-CMs, transforming them from fused network to short rods (Fig. 1E). Electron microscopy analysis revealed that in addition to mitochondrial fragmentation (Fig. 1F, G, I), PINK1 deletion disrupted mitochondrial cristae arrangement, leading to increased intercristal space (Fig. 1F, H). Along with structural abnormalities, mitochondrial function was impaired, as hiPSC-CMs with PINK1 deletion exhibited reduced ΔΨm (Fig. 1J, K) and decreased ATP content (Fig. 1L). However, the generation of reactive oxygen species (ROS) in hiPSC-CMs was not changed by the deletion of PINK1 (Additional file 5: Fig. S3A). Collectively, our findings suggest that inhibition of PINK1 expression disrupts mitochondrial function in hiPSC-CMs and impairs their development, which is not accompanied by ROS accumulation.

PINK1 is a serine/threonine kinase with phosphorylation modifying effects on downstream proteins. To further clarify the role of PINK1 in the developmental maturation of hiPSC-CMs, we used kinetin, a novel activator of PINK1, for its activation. Immunofluorescence results showed that the use of kinetin significantly reduced the disorder of myofibrils arrangement caused by PINK1 deletion (Additional file 6: Fig. S4A). Electron microscopy results showed that the use of kinetin significantly increased the Z-band and sarcomere length of PINK1-deficient hiPSC-CMs (Additional file 6: Fig. S4B–D). Additionally, kinetin reversed the mitochondrial fragmentation of hiPSC-CMs caused by PINK1 deletion (Additional file 6: Fig. S4E). Taken together, these results show that PINK1 is necessary to maintain the mitochondrial quality of hiPSC-CMs and to mediate the development of hiPSC-CMs.

### MS-based quantitative proteomics and characteristics of differentially expressed proteins in PINK1-deficient hiPSC-CMs

**Fig. 2:**
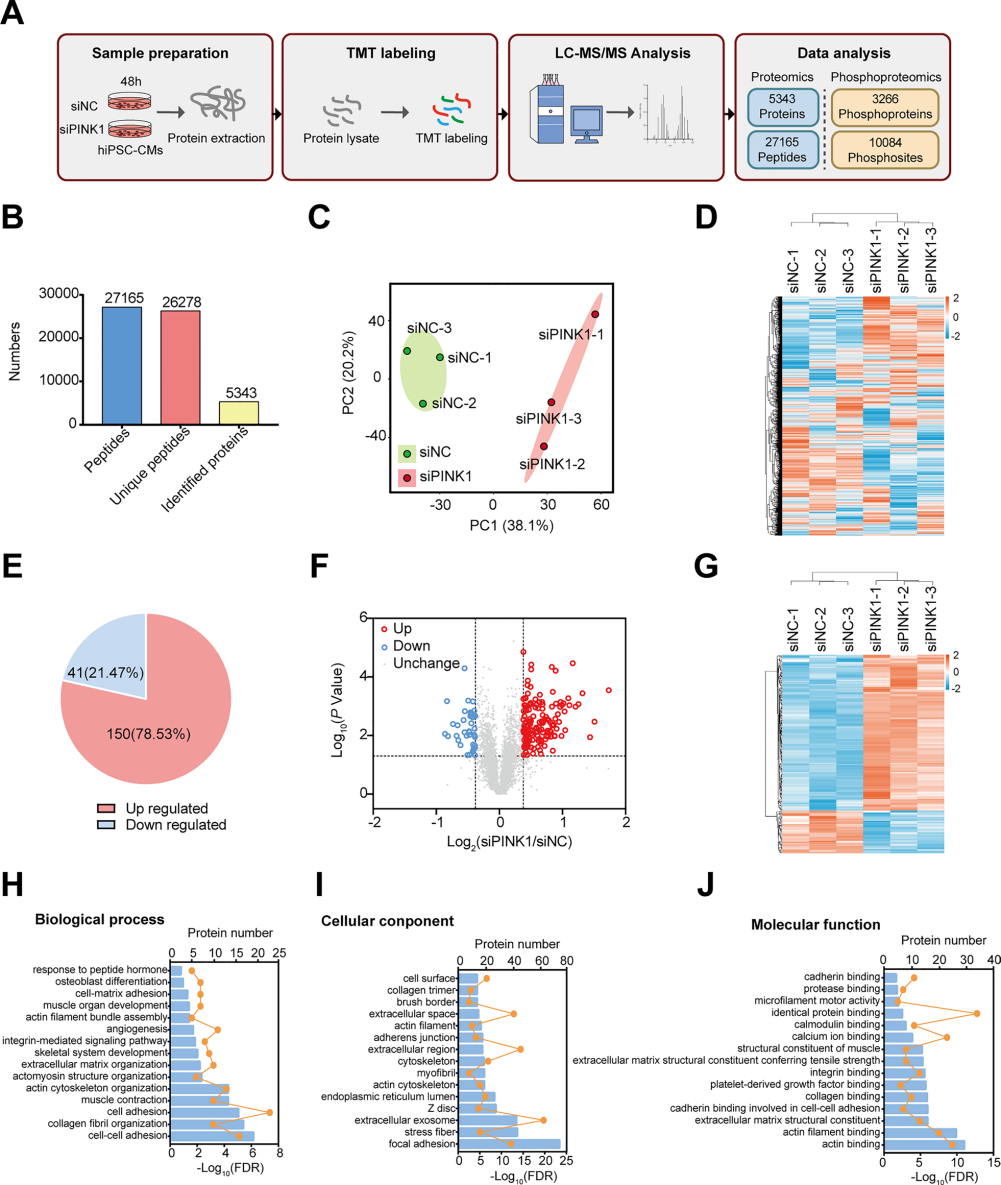
Proteomic analysis of hiPSC-CMs deficient in PINK1. **A** The flow chart of proteomic and phosphoproteomic analysis in this study. **B** Number of peptides, unique proteins, and identified proteins. **C** PCA of proteins identified by proteomic of hiPSC-CMs deficient in PINK1. **D** Hierarchical clustering heatmap of proteins identified by proteomic of hiPSC-CMs deficient in PINK1. **E** Percentage of significantly differentially expressed proteins (*P* < 0.05). **F** Volcano plot of significantly differentially expressed proteins in hiPSC-CMs lacking PINK1. **G** Hierarchical clustering heatmap of significantly differentially expressed proteins in hiPSC-CMs lacking PINK1. **H**–**J** GO enrichment results of significantly differentially expressed proteins identified in hiPSC-CMs lacking PINK1

To gain insight into the molecular mechanism underlying the PINK1 deletion induced disruption of hiPSC-CMs development, MS-based quantitative proteomic was performed on hiPSC-CMs treated with siPINK1 (Fig. 2A). A total of 27,165 peptides and 5343 differentially expressed proteins were identified (Fig. 2B), and Additional file 2: Table S1 provides comprehensive details regarding these proteins. Statistical analysis utilizing principal component analysis (PCA) and hierarchical clustering was employed to visualize the differentially expressed proteins, and the results revealed clear separation between the expression proteins of the siNC group and the siPINK1 group (Fig. 2C, D). These findings suggest that the deletion of PINK1 leads to alterations in the expression levels of numerous proteins in hiPSC-CMs.

To gain further insight into the biological functions of the differentially expressed proteins, we analyzed the significantly differentially expressed proteins identified in the proteomics dataset (*P* < 0.05, FC > 1.3 or FC < 1/1.3). Figure 2E shows 191 proteins that exhibited significant differential expression in the cell lysate protein samples of the siPINK1 group compared to that of the siNC group. Among these, 41 (21.47%) were downregulated proteins, and 150 (78.53%) were upregulated proteins (Fig. 2E). Detailed information about the 191 differentially expressed proteins is provided in Additional file 2: Table S2. The volcano plot in Fig. 2F illustrates the fold changes in protein expression. Additionally, a hierarchical clustering heatmap of the 191 differentially expressed proteins was constructed (Fig. 2G). Subsequently, GO enrichment analysis was performed on these proteins, with a significance threshold of *P* < 0.01, fold enrichment > 2, and a minimum of 5 enriched proteins required for each entry. The top 15 entries in terms of biological process (Fig. 2H), cellular composition (Fig. 2I), and molecular function (Fig. 2J) were screened. The results revealed that these proteins are involved in various biological processes, such as cell adhesion, collagen fiber organization, and cytoskeleton organization. Detailed information about the GO enrichment results from proteomics analysis can be found in Additional file 2: Table S3.

### Phosphoproteomics and characteristics of differentially expressed phosphoproteins in hiPSC-CMs deficient in PINK1

**Fig. 3:**
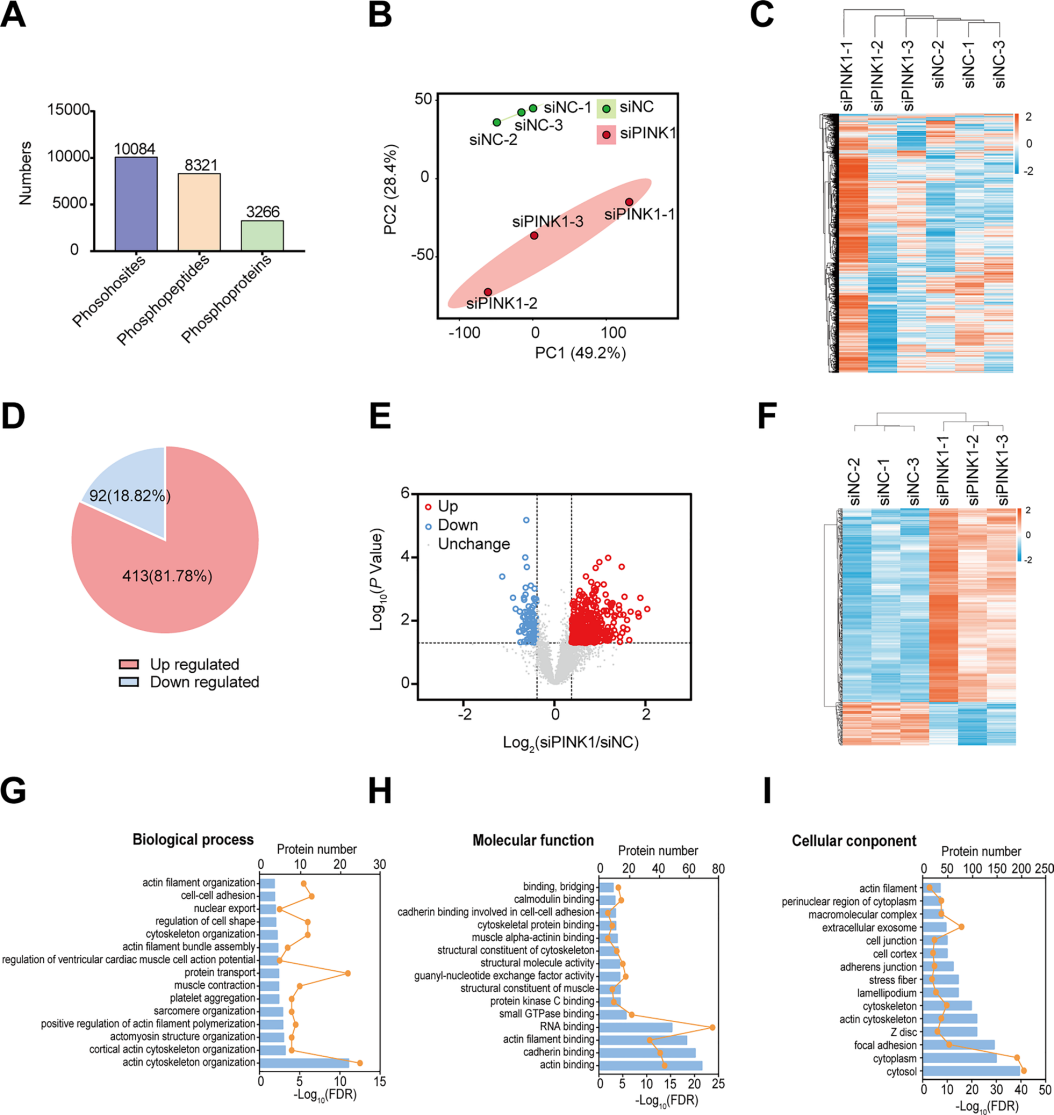
Phosphoproteomic analysis of hiPSC-CMs deficient in PINK1. **A** Number of phosphosites, phosphopeptides, and phosphoproteins. **B** PCA of phosphosites identified by phosphoproteomic in hiPSC-CMs lacking PINK1. **C** Hierarchical clustering heatmap of phosphosites identified by phosphoproteomic in hiPSC-CMs lacking PINK1. **D** Percentage of significantly differentially expressed phosphosites (*P* < 0.05). **E** Volcano map of significantly differentially expressed phosphosites in hiPSC-CMs lacking PINK1. **F** Hierarchical clustering heatmap of significantly differentially expressed phosphosites in hiPSC-CMs lacking PINK1. **G**–**I** GO enrichment analysis of significantly differentially expressed phosphoproteins in hiPSC-CMs lacking PINK1

We postulated that knockdown of PINK1 may impact the phosphorylation levels of downstream proteins. To investigate this, we performed phosphoproteomic profiling and identified 3266 phosphoproteins, 8321 phosphopeptides, and 10,084 phosphosites (Fig. 3A). PCA and a hierarchical clustering analysis heatmap provided a comprehensive overview of the phosphoproteomic data (Fig. 3B, C). The PCA plot of phosphorylated proteomic revealed significant differences between the siPINK1 group and the siNC group. These results indicate that PINK1 deletion leads to altered protein phosphorylation levels in hiPSC-CMs.

We further analyzed the significantly differentially expressed phosphoproteins identified via phosphoproteomic using a significance threshold of *P* < 0.05 and FC > 1.3 or FC < 1/1.3. The siPINK1 group had 347 significant differentially expressed phosphoproteins and 505 significant differentially expressed phosphosites compared to the siNC group. Detailed information about the 505 differentially phosphosites can be found in Additional file 2: Table S4. Among these, 91 (18.82%) phosphosites were found to be hypophosphorylated, and 413 (81.78%) phosphosites were found to be hyperphosphorylated in the siPINK1 group compared to the siNC group (Fig. 3D). A volcano plot (Fig. 3E) and a hierarchical clustering heatmap (Fig. 3F) were generated to visualize the differential expression of phosphosites. Furthermore, GO enrichment analysis of the differentially modified proteins revealed that these proteins were surprisingly localized to the cytoskeleton, and involved in biological processes such as actin cytoskeleton organization, myosin organization, and muscle contraction (Fig. 3G–I). Detailed results of phosphoproteomic GO enrichment analysis can be found in Additional file 2: Table S5.

### Functional diversity of significantly differentially expressed proteins

To further investigate whether there are functional distinctions between up- and downregulated proteins resulting from PINK1 deletion, we conducted separate GO enrichment analyses. We screened the 1152 statistically significant differentially expressed proteins of the 5343 total proteins (*P* < 0.05) and performed GO enrichment analysis based on FC > 1 or FC < 1. Most of the downregulated proteins were localized in mitochondria, the mitochondrial matrix, and the mitochondrial inner membrane (Additional file 7: Fig. S5A), and were associated with biological processes such as mitochondrial translation, energy metabolism, and material transport (Additional file 7: Fig. S5B). Their molecular functions were related to RNA binding (Additional file 7: Fig. S5C). In contrast, the upregulated proteins were localized in focal adhesion, cytosolic exosomes, the endoplasmic reticulum lumen, and the cytoskeleton (Additional file 8: Fig. S6A) and were implicated in cytoskeletal organization and cell adhesion (Additional file 8: Fig. S6B). Their molecular function was associated with actin and cadherin binding (Additional file 8: Fig. S6C).

**Fig. 4:**
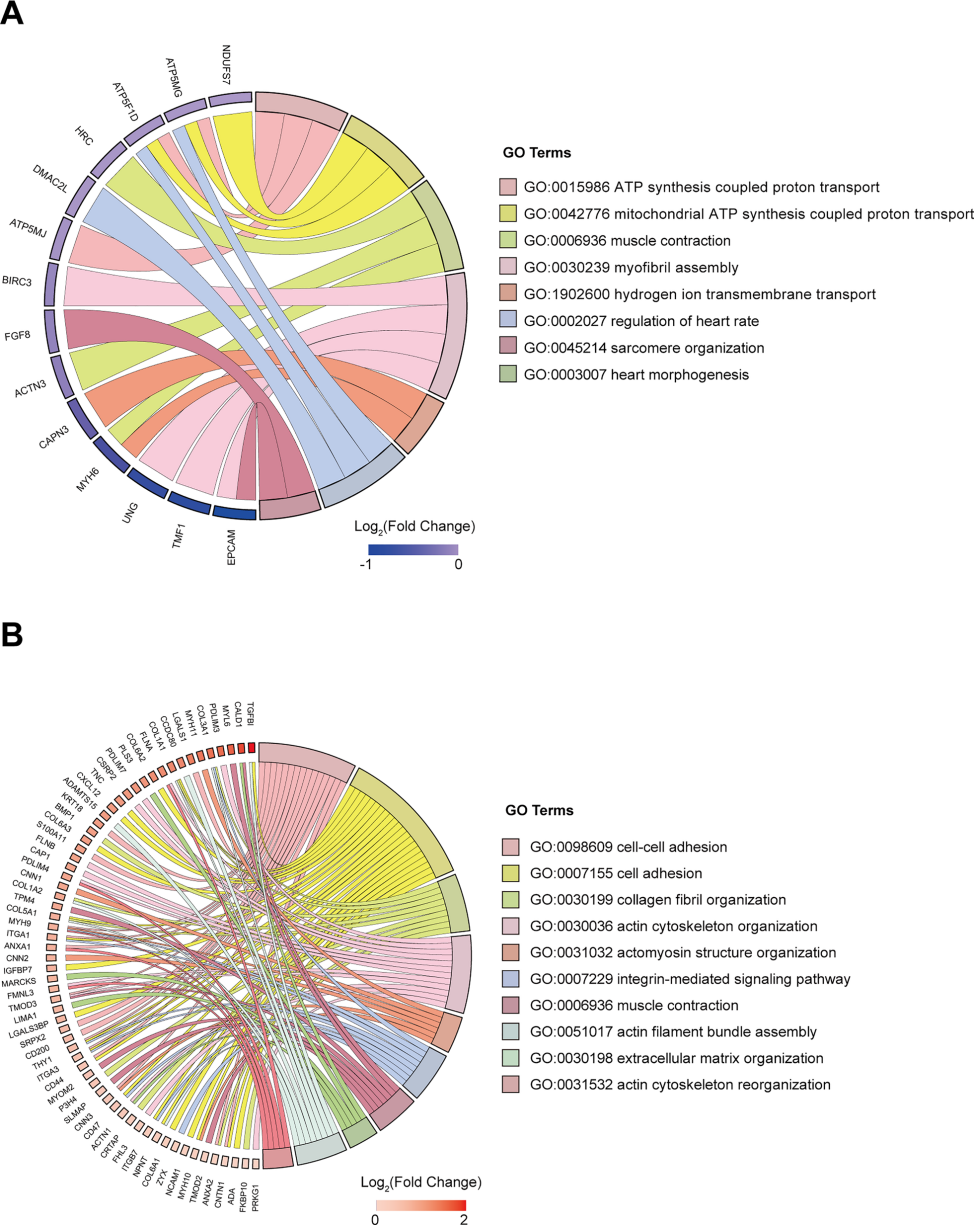
Proteomic identification of significantly differentially expressed proteins in hiPSC-CMs lacking PINK1 involved in different biological processes. **A** GO enrichment result of downregulated proteins in term of biological processes. **B** GO enrichment result of upregulated proteins in term of biological processes

We further found that most of the significantly downregulated proteins (FC < 1/1.3, *P* < 0.05) were involved in ATP synthesis (NDUFS7, ATP5MG, ATP5F1D, ATP5MJ) and muscle contraction (HRC, ACTN3, MYH6) (Fig. 4A). In addition, these proteins were predominantly localized in the mitochondria and Z-band (Additional file 9: Fig. S7A). Meanwhile, the significantly upregulated proteins (FC > 1.3, *P* < 0.05) were primarily associated with cell adhesion and cytoskeleton organization (Fig. 4B) and were mainly localized in the focal adhesion and the cytoskeleton (Additional file 9: Fig. S7C). Furthermore, the molecular functions of these proteins also exhibited differences (Additional file 9: Fig. S7B, D). These results collectively demonstrate that PINK1 deletion leads to differentially expressed proteins in hiPSC-CMs with distinct subcellular localization and functional roles in various biological processes.

**Fig. 5:**
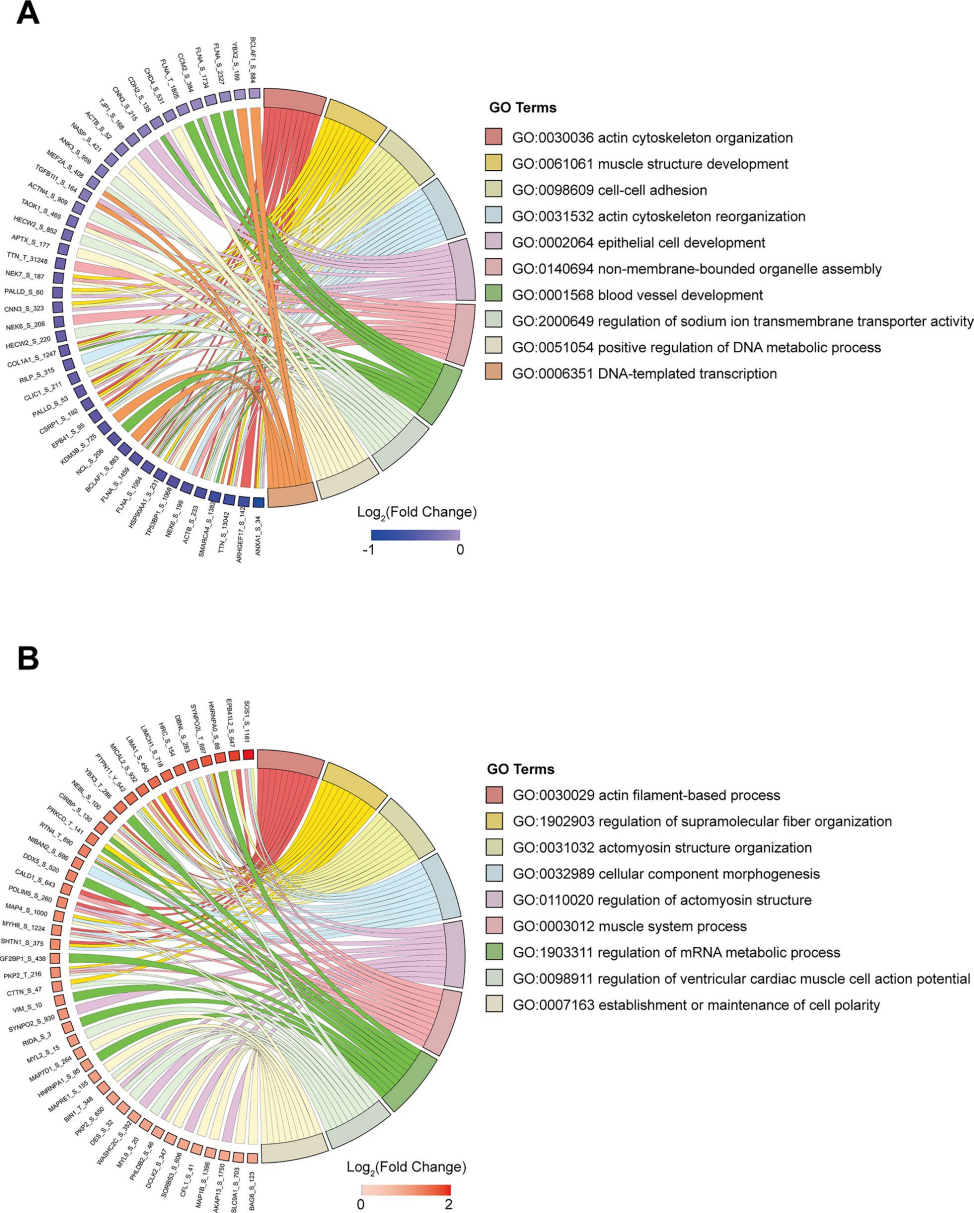
Phosphoproteomic was used to identify differentially expressed phosphoproteins of hiPSC-CMs lacking PINK1 that were involved in different biological processes. **A** GO enrichment result of hypophosphorylated proteins in term of biological processes. **B** GO enrichment result of hyperphosphorylated proteins in term of biological processes

Phosphorylation can alter the function of the cytoskeleton. We hypothesized that PINK1 deletion leads to maturation disorders in hiPSC-CMs, such as disrupted sarcomeres alignment and reduced myocardial contractility, which could be correlated with alterations in actin phosphorylation modifications. Subsequently, we performed GO enrichment analysis of phosphosites with significant upregulations or downregulations of phosphorylation. Figure 5A shows that the significantly downregulated phosphosites (FC < 1/1.3, *P* < 0.05) were predominantly involved in actin cytoskeleton organization and muscle structure. The majority of the significantly upregulated phosphosites (FC > 1.3, *P* < 0.05) were enriched in actin filament-based processes, regulation of supramolecular fiber organization, actomyosin structural organization, and cellular component morphogenesis (Fig. 5B). The functions of these differentially phosphosites were all associated with cytoskeletal organization.

### Identification of signaling pathways and protein interaction network analysis

**Fig. 6:**
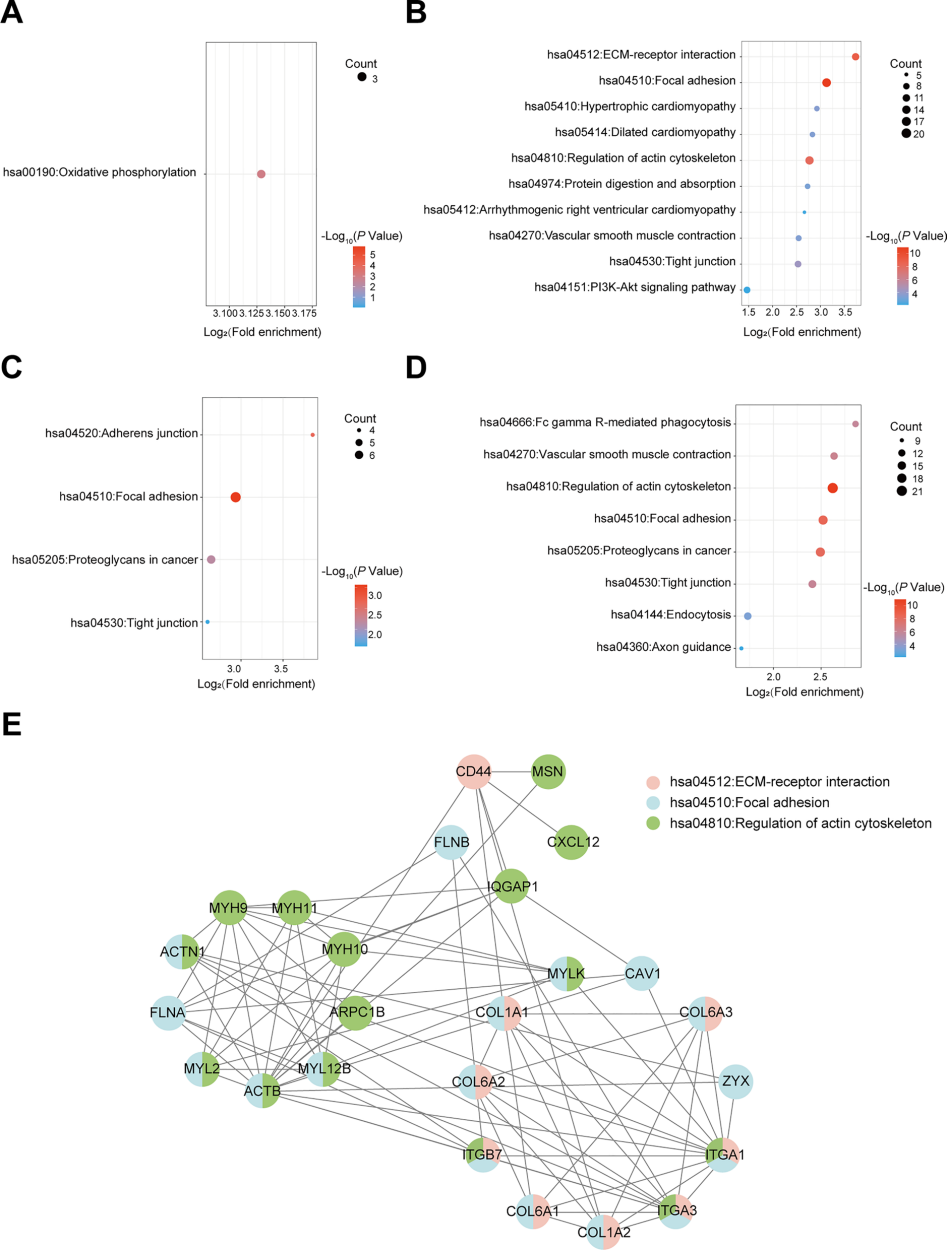
Predicts key signaling pathways and protein interactions of hiPSC-CMs deficient in PINK1. KEGG enrichment results of significantly downregulated protein (**A**) and significantly upregulated protein (**B**). KEGG enrichment results of significantly hypophosphorylated proteins (**C**) and significantly hyperphosphorylated proteins (**D**). **E** Results of protein interaction analysis of significantly upregulated proteins enriched in ECM-receptor interaction, focal adhesion, and regulation of actin cytoskeleton

Given that the significantly differentially expressed proteins were linked to various biological processes, they are likely involved in specific signaling pathways. To explore this further, we conducted KEGG pathway analysis on the proteomic-identified significantly differentially expressed proteins, using a threshold of *P* < 0.05 and FC > 2. Our findings revealed that the significantly downregulated proteins (FC < 1/1.3, *P* < 0.05) were mainly involved in oxidative phosphorylation (Fig. 6A), which is consistent with the biological process enrichment results in Fig. 4A. Moreover, the significantly upregulated proteins (FC > 1.3, *P* < 0.05) showed significant enrichment in three pathways, namely extracellular matrix (ECM)-receptor interaction, focal adhesion, and the regulation of actin cytoskeleton, which were related to cell adhesion and skeletal organization processes as is evident in Fig. 4B. Additionally, some pathways related to cardiac pathological processes were also identified (Fig. 6B). To gain insight into PPI, we conducted protein interaction analysis (confidence = 0.7) using STRING on the proteins enriched in the three aforementioned pathways, and subsequently performed K-means clustering (clusters = 3). Figure 6E. illustrates that ITGA1, ITGA3, and ITGB7 are interconnected and act as intermediate proteins linking the three biological processes. Notably, among these proteins, MYL2, ACTN1, MYLK, COL1A2, COL6A2, and COL6A3 have been associated with dilated cardiomyopathy and hypertrophic cardiomyopathy pathogenic processes in previous studies [22–24].

Subsequently, we conducted KEGG pathway analysis on the differentially expressed phosphoproteins. The results revealed that the hypophosphorylated phosphoproteins (FC < 1/1.3, *P* < 0.05) showed significant enrichment in adherens junction, focal adhesion, and tight junction pathways (Fig. 6C), which correlated with the GO entries shown in Fig. 5A. Furthermore, the hyperphosphorylated phosphoproteins (FC > 1.3, *P* < 0.05) were significantly enriched in the regulation of the actin cytoskeleton pathway (Fig. 6D), as shown in the GO entries in Fig. 5B. The above results indicate that PINK1 deletion significantly inhibited the oxidative phosphorylation pathway of hiPSC-CMs and caused cytoskeletal remodeling.

### Kinase-substrate motif enrichment analysis and upstream kinase prediction

**Fig. 7:**
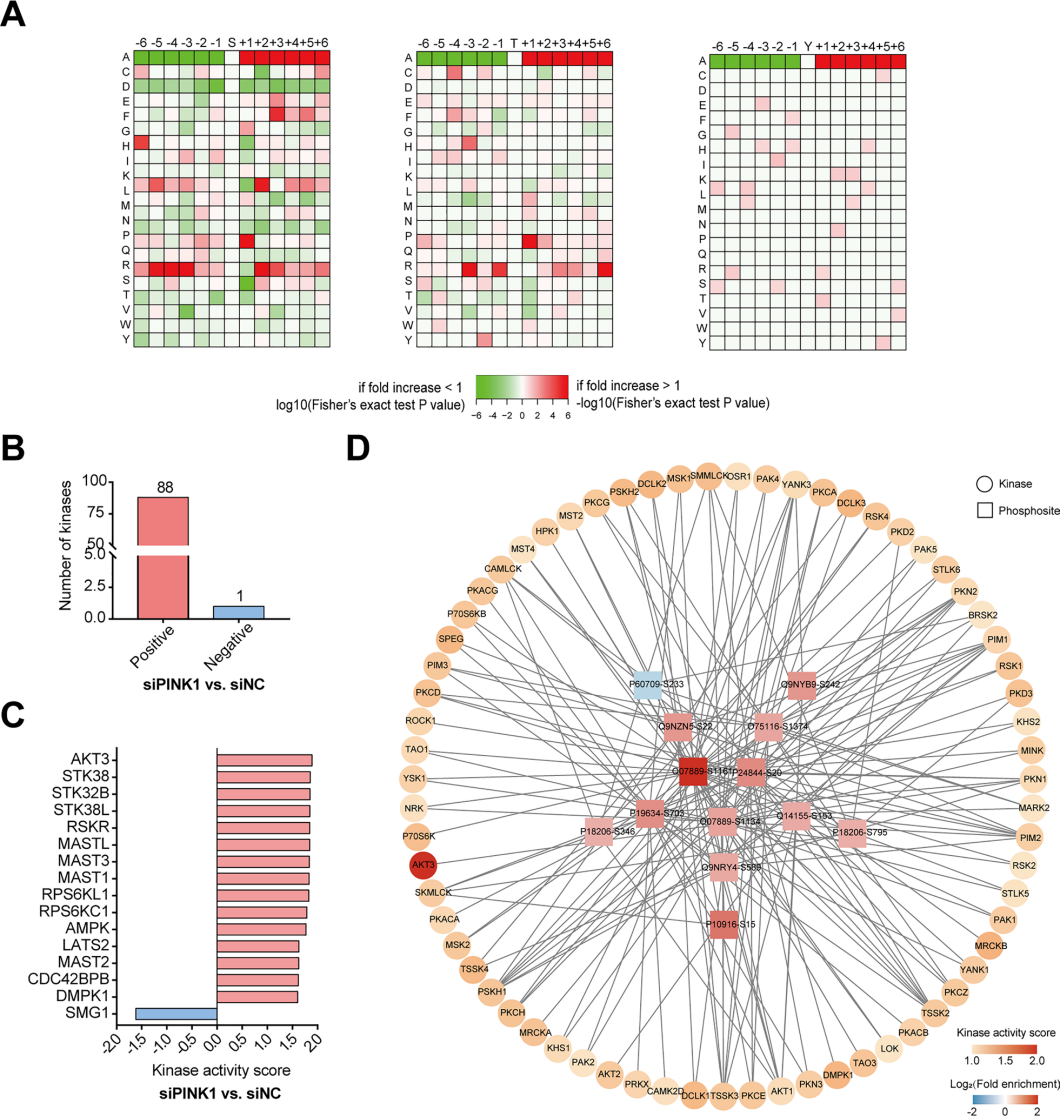
Motif analysis against phosphosites predicts regulatory relationships between upstream kinases and kinase-phosphoproteins. **A** Motif analysis of phosphosites in hiPSC-CMs deficient in PINK1. Predicted number of upstream kinases based on the results of motif analysis (**B**) and kinases with the top 15 kinase activity scores (**C**). **D** Predicted regulatory relationships between kinases and phosphoproteins enriched in regulatory cytoskeletal pathways. The squares represent phosphoproteins; the circles represent upstream kinases. The squares are colored blue for hypophosphorylation and red for hyperphosphorylation; the circles are colored darker or lighter for higher or lower kinase activity scores

In addition, we studied upstream homologous kinases based on iGPS and GSEA methods (*P* < 0.05, FDR < 0.25) with the normalized enrichment score (NES) as the kinase activity score (Fig. 7A). Based on the altered phosphosites in PINK1-deficient hiPSC-CMs, we predicted 89 potential upstream regulated kinases (*P* < 0.05 and FDR < 0.25), including 88 kinases with elevated activity and 1 kinase with decreased activity (Fig. 7B, Additional file 2: Table S6). Figure 7C shows the activity score of the top 15 predicted kinases. Among them, MAST1, MAST2, MAST3 and CDC42BPB were involved in cytoskeleton organization [25]. AMPK respond to changes in the metabolic environment, allowing cells to make adaptive changes [26]. Then, the relationship between enriched genes and kinases in the regulation of the actin cytoskeleton pathway was demonstrated. Figure 7D shows that genes involved in the regulation of the actin cytoskeleton were present in many kinases upstream of the genes involved in the regulation of actin, with the most significant activity being that of AKT3.

### siPINK1 causes abnormalizes the mitochondrial cristae structure of hiPSC-CMs and destabilizes the mitochondrial respiratory chain

Our results showed that the deletion of PINK1 caused an increase in the mitochondrial cristae space in hiPSC-CMs (Fig. 1H). By bioinformatic analysis of the results of proteomic and phosphoproteomic identification, we found that deletion of PINK1 decreased the expression of mitochondrial respiratory chain proteins and inhibited the oxidative phosphorylation pathway in hiPSC-CMs. The respiratory chain complexes is localized in the mitochondrial cristae and is an important complex involved in ATP synthesis [27]. Its structure and function depend on the integrity of the inner mitochondrial membrane, where the efficiency of electron transfer is correlated with the density of the mitochondrial cristae [28–30]. We speculate that PINK1 deletion hinders the development of hiPSC-CMs, probably due to the structural abnormalities of the mitochondrial cristae that destabilize the respiratory chain complexes.

**Fig. 8:**
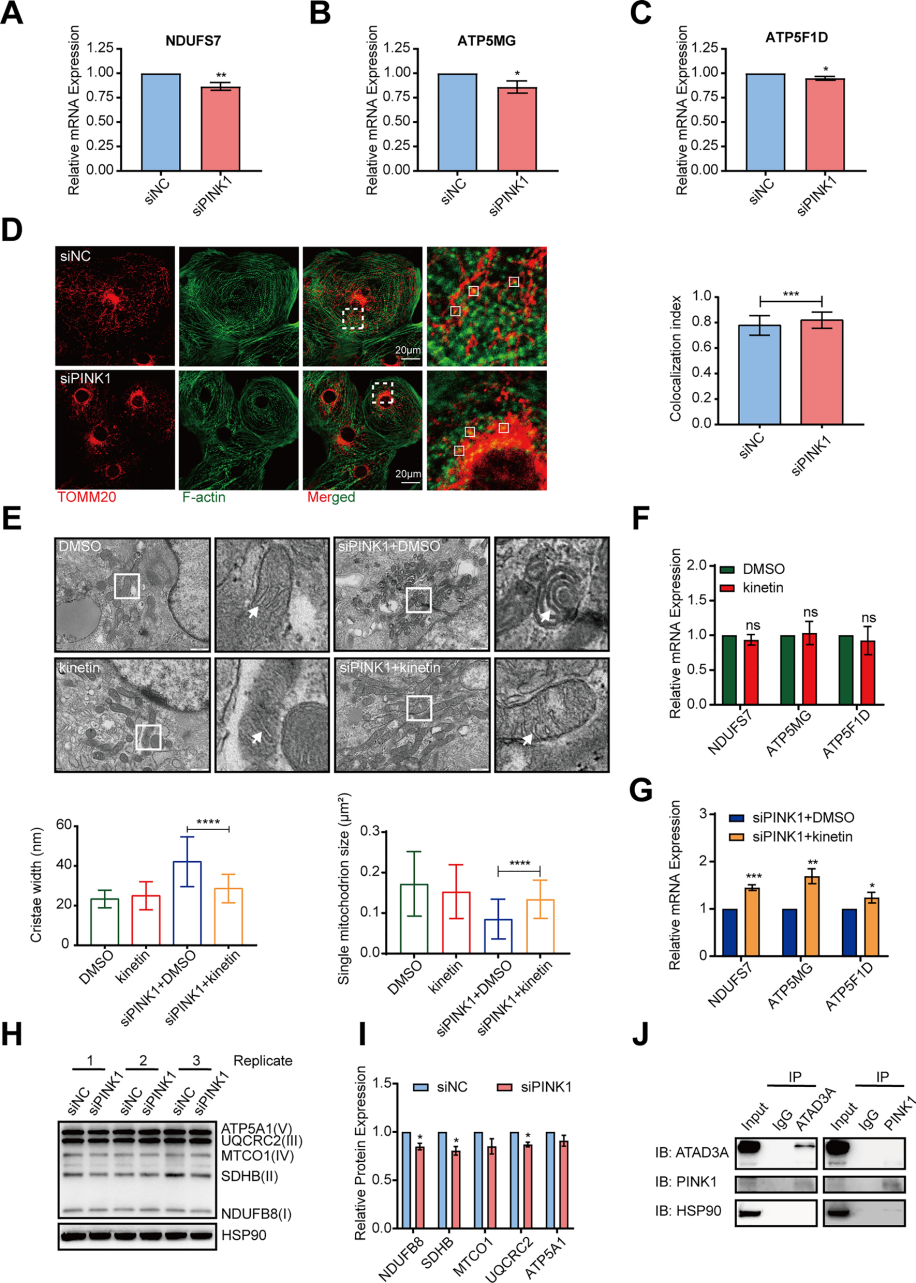
PINK1 deletion disrupts hiPSC-CMs cristae structure and reduces respiratory chain assembly efficiency. **A**–**C** Q-PCR validation of KEGG-enriched genes in oxidative phosphorylation pathways. **D** Immunofluorescence staining of mitochondria and cytoskeleton, ImageJ statistics of Manders overlap coefficient. **E** Electron microscopy images of kinetin treatment of hiPSC-CMs and hiPSC-CMs deficient in PINK1. The white arrows point to the intercristal space of mitochondria. ImageJ was used to examine the cristae width and mitochondrial area. Scale bar = 500 nm. **F**, **G** Q-PCR to detect the mRNA expression levels of NDUFS7, ATP5MG and ATP5F1D after kinetin treatment of hiPSC-CMs and hiPSC-CMs deficient in PINK1. **H** The assembly efficiency of the mitochondrial respiratory chain of hiPSC-CMs lacking PINK1. **I** Statistical plot of the expression of mitochondrial respiratory chain complexes in hiPSC-CMs lacking PINK1. **J** Co-IP detection of the physical binding of PINK1 and ATAD3A. The means ± SEMs are shown. *P < 0.05, **P < 0.01, ***P < 0.001, ****P < 0.0001

To verify this hypothesis, we used q-PCR to confirm the downregulation of proteins involved in oxidative phosphorylation and metabolic pathways, including NDUFS7, ATP5MG, and ATP5F1D, which showed reduced expression in the siPINK1 group compared to the siNC group (Fig. 8A–C). Moreover, we analyzed the localization of mitochondria and cytoskeleton after PINK1 deletion, and our findings revealed an elevated MOC of F-actin and mitochondria, indicating altered mitochondrial-cytoskeleton interactions (Fig. 8D). Electron microscopy results showed that the use of kinetin significantly inhibited mitochondrial fragmentation and increased intercristal spaces in hiPSC-CMs caused by PINK1 deletion (Fig. 8E). Q-PCR results showed that kinetin, although not increasing NDUFS7, ATP5MG, and ATP5F1D expression in hiPSC-CMs in the physiological state (Fig. 8F), significantly increased NDUFS7, ATP5MG, and ATP5F1D expression in hiPSC-CMs lacking PINK1 (Fig. 8G). Meanwhile, western blotting results showed that PINK1 reduced the stability of the respiratory chain complexes (Fig. 8H, I).

To preliminarily investigate the mechanism of PINK1 action in mitochondria, we screened the results of phosphorylation histology identification. We found that PINK1 deletion significantly reduced the serine phosphorylation level at position 632 of ATPase family AAA domain-containing protein 3A (ATAD3A) (FC = 0.7173681, *P* = 0.0065241). ATAD3A is localized in the inner mitochondrial membrane and plays a role in maintaining the structural stability of the inner mitochondrial membrane, cholesterol transport, mitochondrial protein synthesis and mitochondrial DNA copy [31–33]. The Co-IP results showed that there was an interaction between PINK1 and ATAD3A (Fig. 8J). The above results indicate that PINK1 has an important role in the assembly of the respiratory chain complexes in hiPSC-CMs, while ATAD3A is a downstream site for its possible action.

## Discussion

The process of cardiac maturation is accompanied by morphological and functional alterations of mitochondria, and numerous studies have revealed that the mitochondrial dynamics regulator PINK1 has a role in both organism [11] and cardiac development [9, 12, 34]. However, its specific role and molecular mechanisms during myocardial development remain unclear. In this study, quantitative proteomic and phosphoproteomic combined with bioinformatics analysis were used to determine the potential role and mechanism of PINK1 in the developmental maturation of hiPSC-CMs. Our results show that the deletion of PINK1 in hiPSC-CMs resulted in a significant reduction in mitochondrial respiratory chain protein expression and blocked the oxidative phosphorylation pathway. Whereas the expression of proteins associated with cardiac pathology was significantly increased, the phosphorylation levels of proteins involved in cytoskeleton remolding were also significantly altered. We used motif analysis to predict the differentially modified substrates and identified multiple kinases with significantly enhanced activity that play biological roles in cytoskeletal organization and energy metabolism. Finally, we found that PINK1 deletion led to abnormal mitochondrial cristae morphology and impaired mitochondrial respiratory chain assembly in hiPSC-CMs, which may involve ATAD3A. These results may serve as a foundation for studies to explore the role and potential mechanisms of PINK1 in cardiomyocyte development and the intervenable targets of cardiac dysplasia caused by PINK1 deletion.

Fetal cardiomyocytes lack T-tubules, have short and sparse sarcomeres, a low maximal contractility, a high resting potential, absence of T-tubule, and rely mostly on glycolysis for energy [19]. In contrast, mature cardiomyocytes have slim and dense sarcomeres, a lower resting membrane potential, extensive T-tubule formation, and rely on oxidative phosphorylation to produce ATP [35]. Since hiPSC-CMs exhibit structural and functional characteristics similar to those of fetal cardiomyocytes [35, 36], we used them as an in vitro model to investigate the role of PINK1 in cardiomyocyte maturation.

Numerous studies have established that aberrant mitochondrial function is implicated in impaired cardiomyocyte maturation and cardiac pathogenesis [37–39]. Our findings reveal that deletion of PINK1 in hiPSC-CMs causes mitochondrial dysfunction, resulting in disrupted myofibril arrangement and abnormal expression of cytoskeleton, extracellular matrix, and focal adhesion. In contrast, activation of PINK1 reverts the above phenotypes. Previous research has demonstrated that mutations in cytoskeleton and sarcomere genes are associated with dilated cardiomyopathy and hypertrophic cardiomyopathy [22, 23]. Dilated cardiomyopathy is characterized by ECM remodeling and increased expression of focal adhesion proteins [40]. Accumulation of excessive ECM signals through cell surface receptors can lead to myocardial fibrosis and dysfunction [41, 42]. Meanwhile, our kinase prediction results show that AKT3 activity is enhanced in PINK1-deficient hiPSC-CMs. AKT3 is significantly upregulated in the hearts of patients with dilated cardiomyopathy, and AKT3 activation protects the heart from injury; however, persistent activation can cause the heart to dilate or hypertrophy [43, 44]. These findings indicate that PINK1 has a protective function in the heart, and its deletion impairs mitochondrial function and hinders cardiomyocyte development, potentially leading to cardiac disease. Therefore, we suggest that impaired maturation of hiPSC-CMs in the absence of PINK1 may be associated with mitochondrial dysfunction.

The respiratory chain complex is the basic unit of mitochondrial energy supply. Previous studies have demonstrated that PINK1 mutants inhibit respiratory chain complex I activity, block electron transport, and decrease ATP synthesis [9, 45, 46]. In our study, most of the downregulated proteins identified by proteomic were localized in the mitochondria. We observed significant downregulation of proteins such as NDUFS7, ATP5F1D, and ATP5MG, which are involved in the respiratory chain complexes assembly [47, 48]. The respiratory chain complexes are localized to the mitochondrial cristae and organized in the supercomplex for transmembrane proton transfer. The morphology of the mitochondrial cristae is closely related to the assembly of the respiratory chain, and dense mitochondrial cristae exhibit higher respiratory chain complexes activity [28, 30]. Additionally, mitochondria form a greater number of more dense plate-like cristae during cardiac development [49]. Our results demonstrated that PINK1-deficient hiPSC-CMs have increased intercristal space and impaired respiratory chain assembly, while activation of PINK1 reduces the mitochondrial intercristal space. We also found that PINK1 deletion caused hypophosphorylation of ATAD3A-S-632. ATAD3A is localized in mitochondria and is involved in mitochondrial inner membrane assembly [31, 32]. We further found that PINK1 and ATAD3A bind to each other, while the effect of hypophosphorylated ATAD3A on mitochondrial cristae morphology and the specific mechanism of ATAD3A regulation by PINK1 need to be further investigated. In summary, we suggest that PINK1 deficiency causes abnormal mitochondrial cristae morphology in hiPSC-CMs as the reason for inhibiting respiratory chain assembly and hindering energy production.

Due to its high metabolic capacity, the heart is particularly susceptible to oxidative stress, and mutations in PINK1 have been shown to increase ROS production [9, 50, 51]. Prolonged exposure to ROS has been implicated in the development of cardiac hypertrophy [52], ultimately leading to heart failure. Previous studies have demonstrated that endothelial cells respond to oxidative stress by rapidly generating focal adhesion and activating MAPKAP kinase 2/3 to phosphorylate actin, resulting in rapid reorganization and ultimately the destruction of cortical microfilaments [53]. Additionally, prostaglandin E2 has been shown to promote ROS production in keratinocytes [54], leading to the phosphorylation of ROCK-S-1379 and the disruption of characteristic actin fibers in fibroblasts [55]. However, our experimental results did not reveal an increase in ROS production upon PINK1 deletion. This shows that PINK1 deletion causes impaired maturation of hiPSC-CMs independent of oxidative stress injury.

The morphological changes in cardiomyocytes during heart development involve cardiomyocyte proliferation to increase heart volume prenatally and increased cardiomyocyte volume to allow further growth postnatally [56]. Cardiomyocyte morphology and function are predominantly influenced by the remodeling of the actin cytoskeleton [57, 58]. Actin dynamics can be regulated through post-translational modifications (PTMs), such as phosphorylation, which induces rapid cytoskeletal remodeling in neuronal actin; meanwhile, dephosphorylation stabilizes actin filaments [59]. PINK1 is a serine/threonine kinase that exerts phosphorylation modifications through its C-terminal structural domain [60–62]. In our study, we observed that the deletion of PINK1 altered the phosphorylation of numerous actin proteins, although PINK1 was not identified among the predicted upstream kinases. This may be PINK1 belongs to a distinct family in the kinase tree [63], and limited research on its downstream substrates has been performed, which could explain its absence from the list of predicted kinases.

The function of mitochondria depends on their morphology and distribution, which involves the cytoskeleton [64]. Previous studies have demonstrated that actin is localized to specific mitochondrial subpopulations and plays a dynamic role in regulating mitochondrial motility and depolymerization [65, 66]. Additionally, actin has been shown to respond to impaired oxidative phosphorylation caused by defects in the mitochondrial respiratory chain, thereby promoting cell survival [67]. Notably, ATP, which accounts for nearly 50% of cellular energy, plays a crucial role in maintaining the structural integrity of actin and facilitating cytoskeleton remodeling [68]. Our findings reveal that PINK1 deletion results in mitochondrial fragmentation and dysfunction in hiPSC-CMs, accompanied by increased colocalization of the mitochondria with the cytoskeleton, suggesting a complex dynamic regulatory relationship between the mitochondria and the actin skeleton. AMP-activated protein kinase (AMPK), a key energy sensor [69], is involved in cellular actin skeleton rearrangement in response to external mechanical stimuli [70–72]. Our predicted kinase results indicate enhanced AMPK activity, which may be a response to the energy crisis caused by PINK1 deletion. However, further experimental evidence is required to determine whether PINK1 deletion leads to altered levels of cytoskeletal phosphorylation in hiPSC-CMs as an adaptation to insufficient mitochondrial energy supply.

## Conclusions

In conclusion, our study highlights the crucial role of PINK1 as an essential regulator in the maturation of hiPSC-CMs, as it directly affects respiratory chain assembly and contributes to maintaining the production of energy required for cardiomyocyte development. The deletion of PINK1 may result in mitochondrial dysfunction and reduced energy supply, triggering adaptive remodeling of the actin cytoskeleton in hiPSC-CMs, ultimately leading to abnormal development. These findings provide a foundation for further investigation into the complex relationship between PINK1, a mitochondrial protein involved in regulating mitochondrial function in cardiomyocytes, and cytoskeletal remodeling during myocardial development.

### Supplementary Information

**Additional file 1.**

**Additional file 2: Table S1.** Details of 5343 differentially expressed proteins identified by proteomic. **Table S2.** Details of 191 significantly differentially expressed proteins identified by proteomic. **Table S3.** 191 GO enrichment details of significantly differentially expressed proteins. **Table S4.** Details of 505 significantly different phosphorsites identified by phosphoproteomic. **Table S5.** 437 GO enrichment details of significantly differentially expressed phosphoproteins. **Table S6.** GSEA kinase prediction results.

**Additional file 3: Figure S1.** Culture and identification of hiPSCs and hiPSC-CMs. (A) Flow chart of hiPSC differentiation into hiPSC-CMs. (B) Immunofluorescence identification of hiPSCs expressing NANOG, SOX2, and OCT4 stemness markers in hiPSCs. (C) Immunofluorescence identification of hiPSC-CMs expressing α-actinin, TNNT2, and CX43 myocardial markers. (D) Ultrastructure of hiPSCs and hiPSC-CMs. Mitochondria (red arrows), Z-band (blue arrows), sarcomere (yellow arrows).

**Additional file 4: Figure S2.** Western blotting identification of PINK1 expression in hiPSC-CMs deficient in PINK1.

**Additional file 5: Figure S3.** DCFH staining of hiPSC-CMs deficient in PINK1.

**Additional file 6: Figure S4.** Activation of PINK1 prevents the impaired maturation of hiPSC-CMs caused by PINK1 deletion. (A) α-actinin staining of kinetin treatment of hiPSC-CMs and after deletion of PINK1 in hiPSC-CMs. α-actinin (green). Scale bar = 20 μm. (B) Electron microscopy images of kinetin treatment of hiPSC-CMs and hiPSC-CMs deficient in PINK1. ImageJ assay Z-band width (C) and sarcomere length (D). Scale bar = 1 μm. (E) MitoTracker staining of kinetin treatment of hiPSC-CMs and after deletion of PINK1 in hiPSC-CMs. MitoTracker (green). Scale bar = 20 μm. The means ± SEMs are shown. **P < 0.01, ***P < 0.001.

**Additional file 7: Figure S5.** Proteomic identification of hiPSC-CMs deficient in PINK1 for significantly differentially expressed proteins in cellular components and molecular functions of GO enrichment. (A) GO enrichment result of significantly downregulated proteins in terms of cellular composition and (B) molecular function. (C) GO enrichment result of significantly upregulated proteins in terms of cellular composition and (D) molecular functional.

**Additional file 8: Figure S6.** Proteomic identification of GO enrichment results of downregulated expressed proteins in hiPSC-CMs deficient in PINK1. (A) GO enrichment result of downregulated proteins in terms of cellular composition, (B) biological processes and (C) molecular functions.

**Additional file 9: Figure S7.** Proteomic identification of GO enrichment results of upregulated proteins in hiPSC-CMs deficient in PINK1. (A) GO enrichment results of upregulated proteins in terms of cellular composition, (B) biological processes and (C) molecular functions.

## Data Availability

The datasets used and/or analyzed during the current study are available from the corresponding author on reasonable request.

## Abbreviations

hiPSC-CMs: Human induced pluripotent stem cell-derived cardiomyocytes
hiPSCs: Human induced pluripotent stem cells
PINK1: PTEN-inducible kinase 1
FDR: False discovery rate
ΔΨm: Mitochondrial membrane potential
MOC: Manders overlap coefficient
PCA: Principal component analysis
FC: Fold change
NES: Normalized enrichment score
ECM: Extracellular matrix
PTMs: Post-translational modifications
ROS: Reactive oxygen species
AMPK: AMP-activated protein kinase
KEGG: Kyoto Encyclopedia of Genes and Genomes
DAVID: Database for annotation, visualization and integrated discovery
