# Supplementary figures and images for: Quantitative proteomic and phosphoproteomic analysis reveal the relationship between mitochondrial dysfunction and cytoskeletal remodeling in hiPSC-CMs deficient in PINK1
Source: J Transl Med. 2023 Aug 30;21:581. doi: 10.1186/s12967-023-04467-y (PMC10466879; doi:10.1186/s12967-023-04467-y)

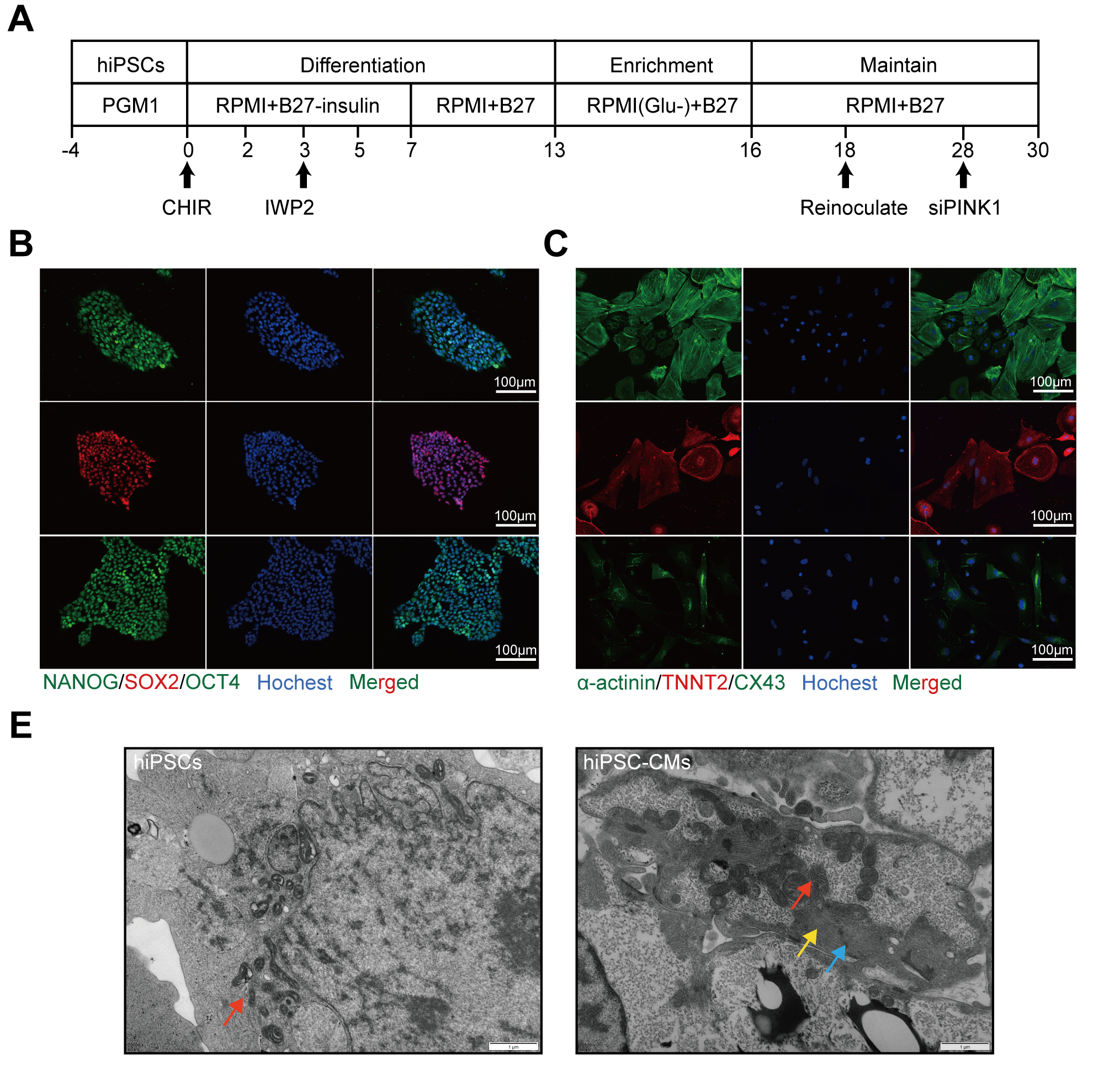

Supplement: Supplementary file 3 — Additional file 3: Figure S1. Culture and identification of hiPSCs and hiPSC-CMs. (A) Flow chart of hiPSC differentiation into hiPSC-CMs. (B) Immunofluorescence identification of hiPSCs expressing NANOG, SOX2, and OCT4 stemness markers in hiPSCs. (C) Immunofluorescence identification of hiPSC-CMs expressing α-actinin, TNNT2, and CX43 myocardial markers. (D) Ultrastructure of hiPSCs and hiPSC-CMs. Mitochondria (red arrows), Z-band (blue arrows), sarcomere (yellow arrows). [file 12967_2023_4467_MOESM3_ESM.tif]

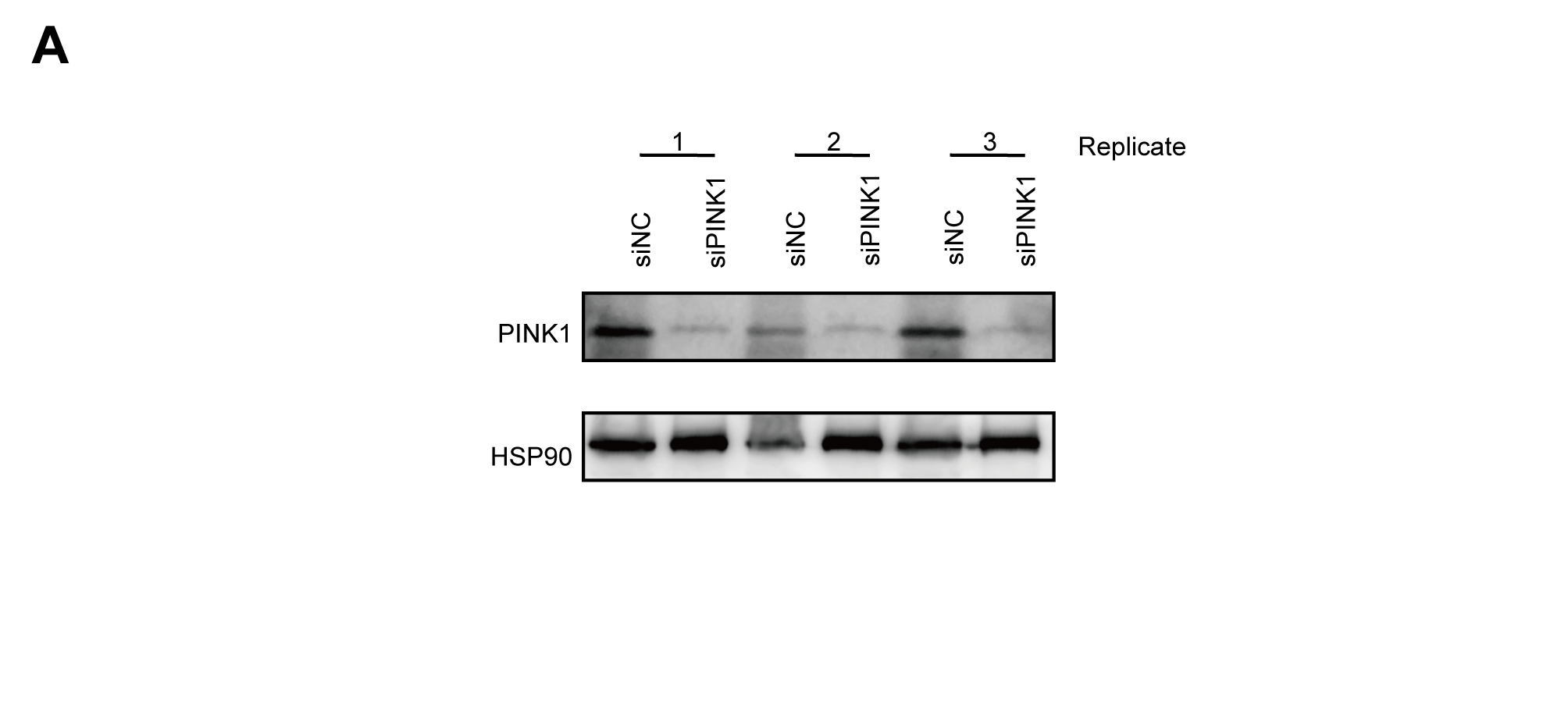

Supplement: Supplementary file 4 — Additional file 4: Figure S2. Western blotting identification of PINK1 expression in hiPSC-CMs deficient in PINK1. [file 12967_2023_4467_MOESM4_ESM.tif]

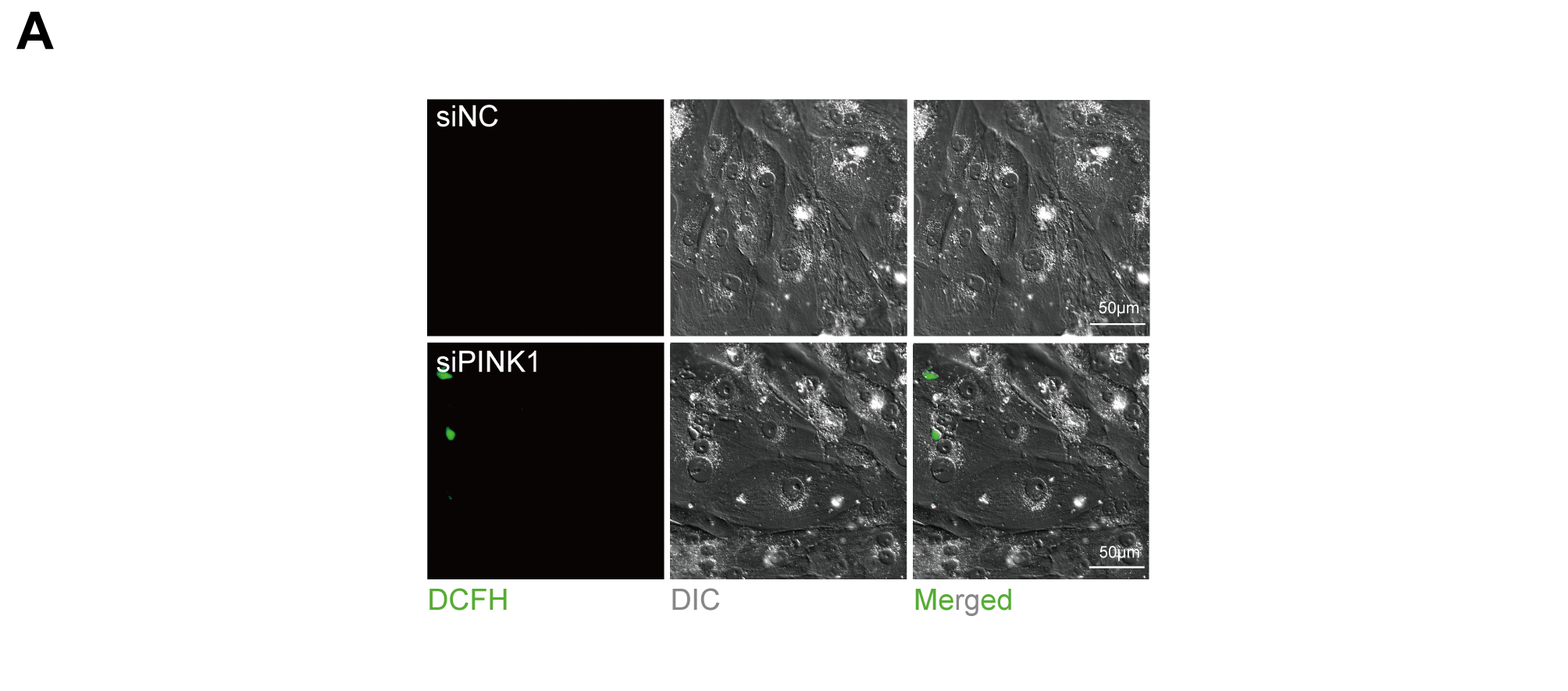

Supplement: Supplementary file 5 — Additional file 5: Figure S3. DCFH staining of hiPSC-CMs deficient in PINK1. [file 12967_2023_4467_MOESM5_ESM.tif]

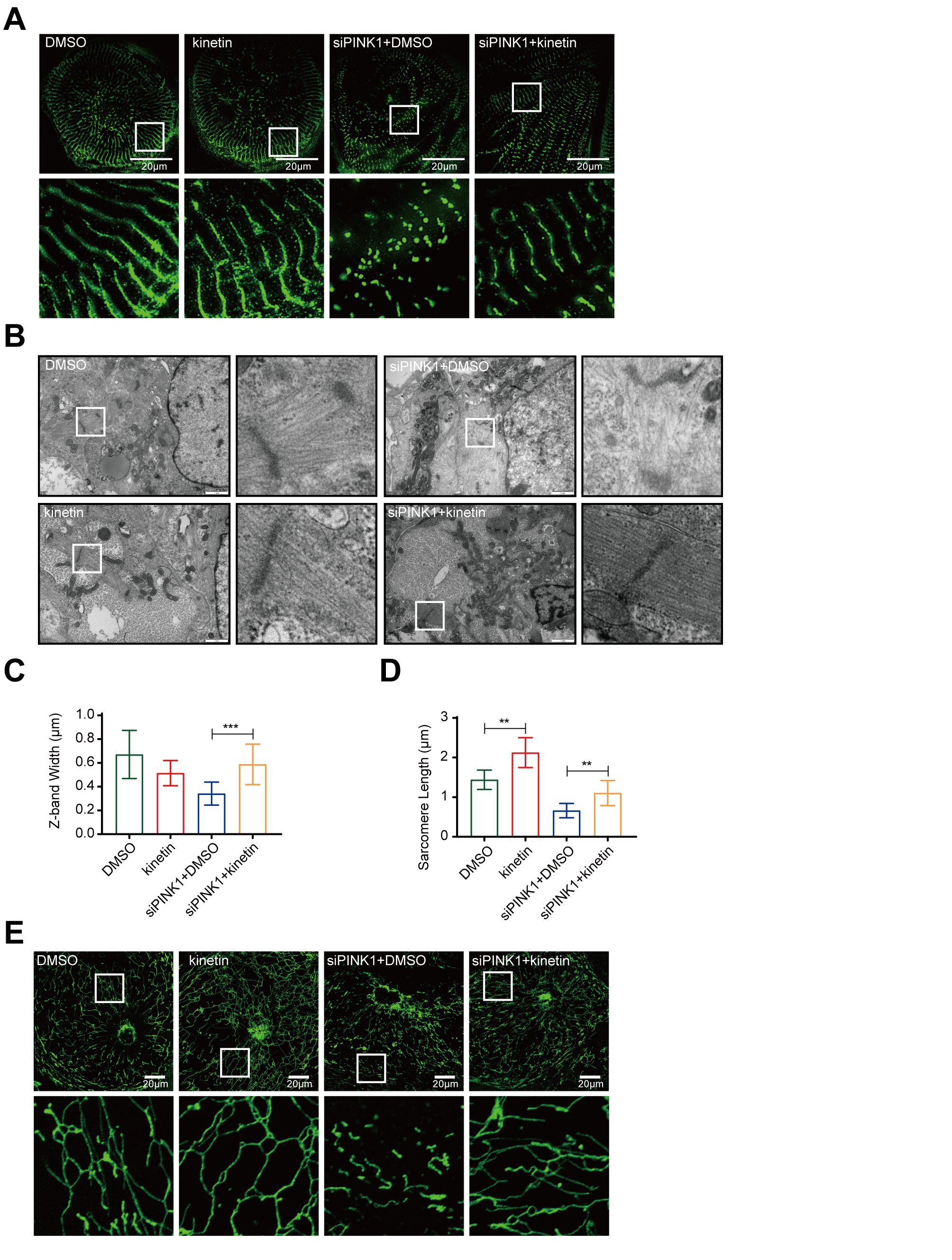

Supplement: Supplementary file 6 — Additional file 6: Figure S4. Activation of PINK1 prevents the impaired maturation of hiPSC-CMs caused by PINK1 deletion. (A) α-actinin staining of kinetin treatment of hiPSC-CMs and after deletion of PINK1 in hiPSC-CMs. α-actinin (green). Scale bar = 20 μm. (B) Electron microscopy images of kinetin treatment of hiPSC-CMs and hiPSC-CMs deficient in PINK1. ImageJ assay Z-band width (C) and sarcomere length (D). Scale bar = 1 μm. (E) MitoTracker staining of kinetin treatment of hiPSC-CMs and after deletion of PINK1 in hiPSC-CMs. MitoTracker (green). Scale bar = 20 μm. The means ± SEMs are shown. **P < 0.01, ***P < 0.001. [file 12967_2023_4467_MOESM6_ESM.tif]

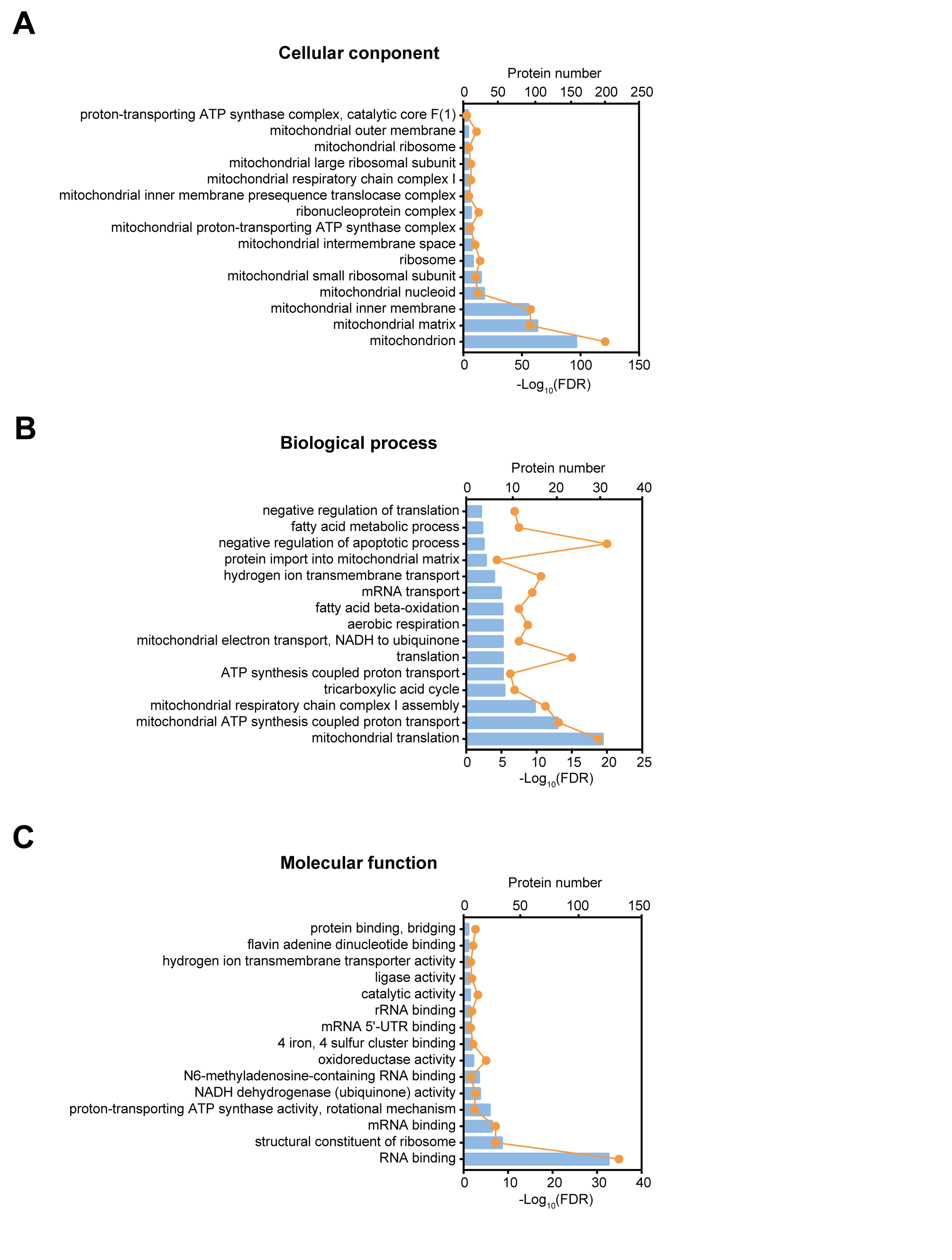

Supplement: Supplementary file 7 — Additional file 7: Figure S5. Proteomic identification of hiPSC-CMs deficient in PINK1 for significantly differentially expressed proteins in cellular components and molecular functions of GO enrichment. (A) GO enrichment result of significantly downregulated proteins in terms of cellular composition and (B) molecular function. (C) GO enrichment result of significantly upregulated proteins in terms of cellular composition and (D) molecular functional. [file 12967_2023_4467_MOESM7_ESM.tif]

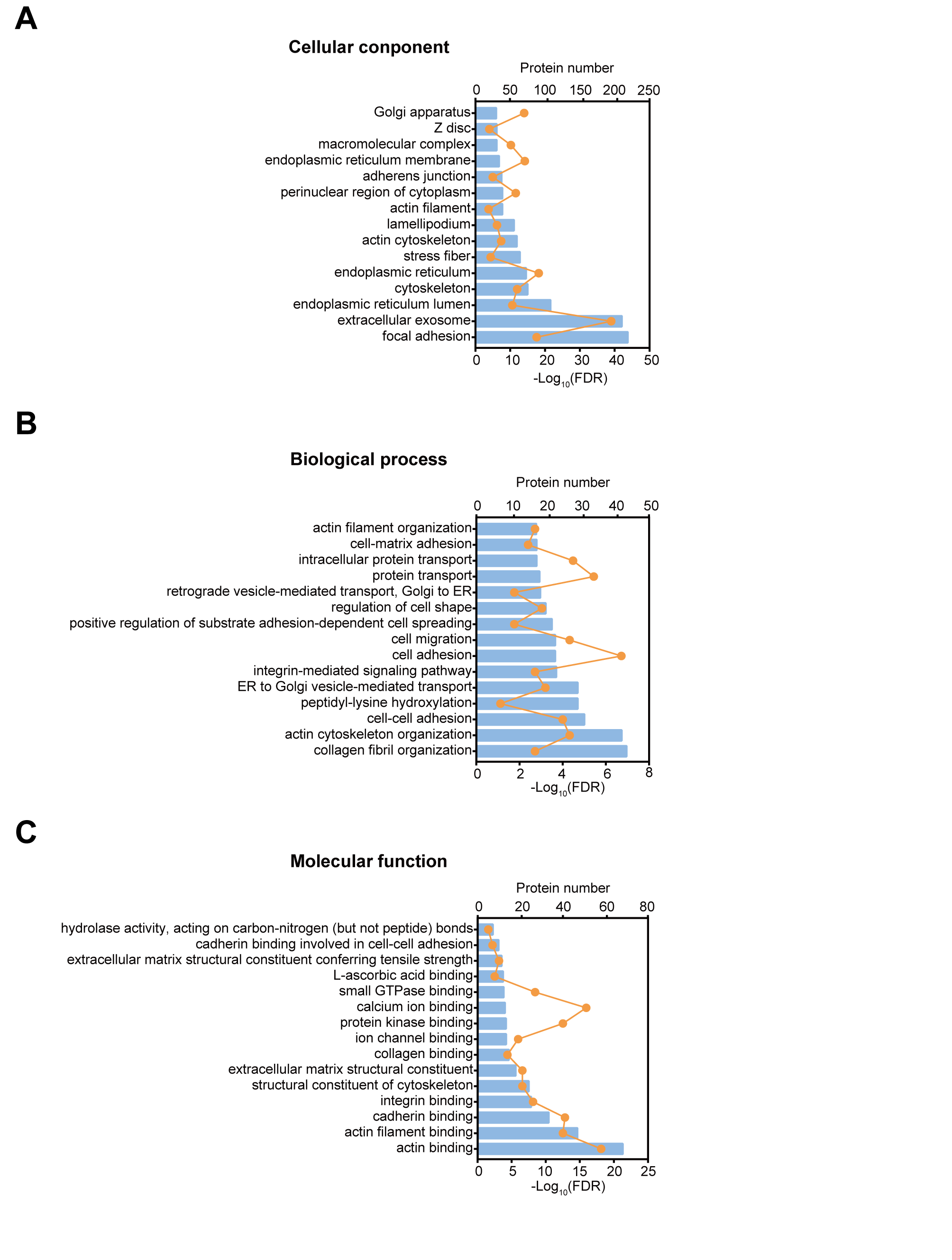

Supplement: Supplementary file 8 — Additional file 8: Figure S6. Proteomic identification of GO enrichment results of downregulated expressed proteins in hiPSC-CMs deficient in PINK1. (A) GO enrichment result of downregulated proteins in terms of cellular composition, (B) biological processes and (C) molecular functions. [file 12967_2023_4467_MOESM8_ESM.tif]

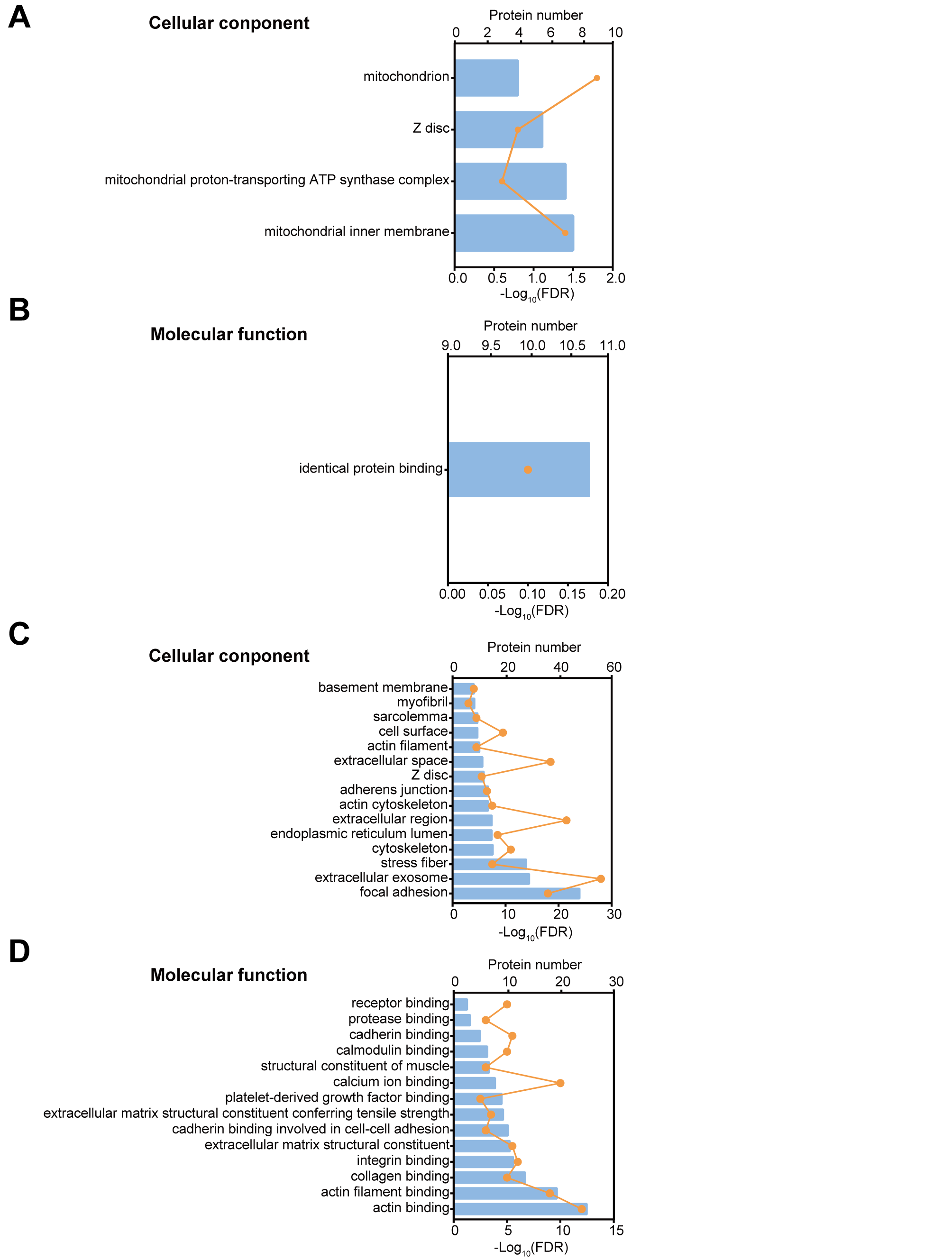

Supplement: Supplementary file 9 — Additional file 9: Figure S7. Proteomic identification of GO enrichment results of upregulated proteins in hiPSC-CMs deficient in PINK1. (A) GO enrichment results of upregulated proteins in terms of cellular composition, (B) biological processes and (C) molecular functions. [file 12967_2023_4467_MOESM9_ESM.tif]
